# Long-Fiber Carbon Nanotubes Replicate Asbestos-Induced Mesothelioma with Disruption of the Tumor Suppressor Gene *Cdkn2a* (*Ink4a/Arf*)

**DOI:** 10.1016/j.cub.2017.09.007

**Published:** 2017-11-06

**Authors:** Tatyana Chernova, Fiona A. Murphy, Sara Galavotti, Xiao-Ming Sun, Ian R. Powley, Stefano Grosso, Anja Schinwald, Joaquin Zacarias-Cabeza, Kate M. Dudek, David Dinsdale, John Le Quesne, Jonathan Bennett, Apostolos Nakas, Peter Greaves, Craig A. Poland, Ken Donaldson, Martin Bushell, Anne E. Willis, Marion MacFarlane

**Affiliations:** 1 Medical Research Council Toxicology Unit, Hodgkin Building, PO Box 138, Lancaster Road, Leicester LE1 9HN, UK; 2 Medical Research Council/University of Edinburgh, Centre for Inflammation Research, Queen's Medical Research Institute, Edinburgh EH16 4TJ, UK; 3 University Hospitals of Leicester NHS Trust, Glenfield Hospital, Leicester LE3 9QP, UK; 4 Department of Cancer Studies, University of Leicester, Leicester LE2 7LX, UK

**Keywords:** asbestos, mesothelioma, carbon nanotubes, tumor suppressor genes, epigenetics, hypermethylation, *CDKN2A*, toxicity

## Abstract

Mesothelioma is a fatal tumor of the pleura and is strongly associated with asbestos exposure. The molecular mechanisms underlying the long latency period of mesothelioma and driving carcinogenesis are unknown. Moreover, late diagnosis means that mesothelioma research is commonly focused on end-stage disease. Although disruption of the *CDKN2A* (*INK4A/ARF*) locus has been reported in end-stage disease, information is lacking on the status of this key tumor suppressor gene in pleural lesions preceding mesothelioma. Manufactured carbon nanotubes (CNTs) are similar to asbestos in terms of their fibrous shape and biopersistent properties and thus may pose an asbestos-like inhalation hazard. Here we show that instillation of either long CNTs or long asbestos fibers into the pleural cavity of mice induces mesothelioma that exhibits common key pro-oncogenic molecular events throughout the latency period of disease progression. Sustained activation of pro-oncogenic signaling pathways, increased proliferation, and oxidative DNA damage form a common molecular signature of long-CNT- and long-asbestos-fiber-induced pathology. We show that hypermethylation of *p16/Ink4a* and *p19/Arf* in CNT- and asbestos-induced inflammatory lesions precedes mesothelioma; this results in silencing of *Cdkn2a* (*Ink4a/Arf*) and loss of p16 and p19 protein, consistent with epigenetic alterations playing a gatekeeper role in cancer. In end-stage mesothelioma, silencing of *p16/Ink4a* is sustained and deletion of *p19/Arf* is detected, recapitulating human disease. This study addresses the long-standing question of which early molecular changes drive carcinogenesis during the long latency period of mesothelioma development and shows that CNT and asbestos pose a similar health hazard.

## Highlights

• Long-fiber carbon nanotubes induce malignant mesothelioma with loss of *Cdkn2a*
• Long-fiber carbon nanotube tumors replicate asbestos-induced mesothelioma in humans
• *Cdkn2a* hypermethylation during disease latency precedes fiber-induced mesothelioma
• Long-fiber carbon nanotubes pose an asbestos-like hazard

## Teaser

Manufactured carbon nanotubes (CNTs) show structural similarities to asbestos, strongly associated with the fatal tumor mesothelioma. Chernova et al. show that CNTs and asbestos indeed pose similar health hazards and address the long-standing question of which early molecular changes drive carcinogenesis during mesothelioma’s long latency period.

## Introduction

Malignant mesothelioma is an aggressive, incurable tumor strongly associated with asbestos exposure and with increasing incidence reported worldwide. Length-dependent retention of asbestos fibers in the pleural cavity is crucial for disease development, with chronic inflammation playing an important role in carcinogenesis [1, 2]. The latency period of this disease extends to 40 years, and molecular events leading to malignant transformation are poorly understood. Mesothelioma in humans is considered to be a “disease of gene loss,” rather than being associated with driver mutations [3]. Genetic analyses identified several key genetic alterations in end-stage disease, with most common deletions or mutations in *CDKN2A*, *NF2*, and *BAP1* genes [4], and suggested that two main pathways, p53/DNA repair and PI3K-AKT, were associated with mesothelioma progression [5]. Compromised antioxidant response due to mutation of DNA repair genes may also contribute to oncogenesis [6]. Epigenetic alterations play a gatekeeper role in cancer as they are the earliest observable genetic change [7]. DNA hypermethylation is commonly associated with tumor suppressor gene silencing. Transcriptional inactivation of these genes and the loss of their functions most frequently occur during the early stages of the carcinogenesis and at the pre-cancerous stages of tumor [7, 8]. Although disruption of the *CDKN2A* locus, including *p16/INK4A* silencing, has been reported in end-stage disease [4, 9], the key molecular events that occur during the long latency period of mesothelioma are unknown. Due to their high aspect ratio and biopersistent properties, carbon nanotubes (CNTs) may pose an asbestos-like inhalation hazard [10, 11, 12]. Several thousand tons of CNTs are produced each year, and these new compounds have many commercial and medical applications, including their incorporation into sports equipment, computers, and building materials. *In vivo* studies have indicated that CNTs can cause sustained inflammation and fibrosis of the pleura [1] and can also induce tumor development; however, this has only been shown in genetically susceptible or peritoneally exposed rodent models [12, 13, 14, 15]. Data comparing prolonged pleural exposure of wild-type animals to occupationally relevant doses of CNTs or asbestos is lacking, and the molecular mechanisms underlying fiber-induced carcinogenesis have not been explored [16].

Here we investigate the effect of instillation of long CNTs and long asbestos into the pleural cavity of mice, the major site of tumor development in humans. We show that long CNTs and long asbestos induce mesothelioma with deletion of *p19/Arf* and silencing of *p16/Ink4a*, highlighting that epigenetic alterations play a gatekeeper role in mesothelioma. Importantly, this study identifies, for the first time, key molecular events underlying progression of long-CNT-induced inflammatory lesions to malignant mesothelioma and addresses the long-standing question of which molecular changes drive carcinogenesis during the latency period of asbestos-induced mesothelioma.

## Results

### Common Molecular Signature of Inflammatory Lesions Induced by Asbestos and CNTs

In three parallel studies, we exposed mice to low, occupationally relevant doses of CNTs, consecutively reducing the dose and increasing exposure time: 5 μg/mouse for 1–12 weeks; 2.5 μg for 1 year; and 1 μg, 0.5 μg, and 0.2 μg for up to 20 months. In each study, a comparator group of animals was subjected to 25 μg amosite asbestos fiber exposure. Long (pathogenic) and short (not associated with disease) fiber amosite asbestos (LFA and SFA, respectively; [17, 18]) and long and short CNTs (LNTs and SNTs, respectively) were injected into the pleural cavity of mice, the major site of mesothelioma development (Figure S1 and STAR Methods), and the responses along the pleura were assessed at time points up to 20 months post-injection. This approach reflects the localization of fibers in the pleural space observed after translocation of fibers from the lungs [19].

**Figure 1:**
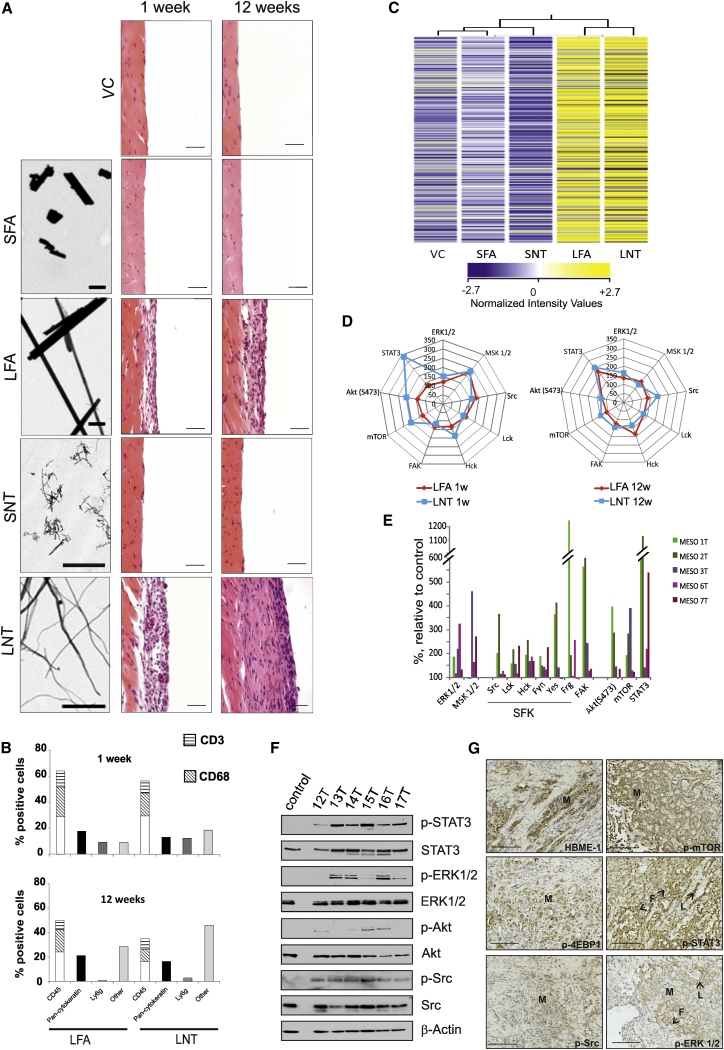
Asbestos and CNT Fiber-Induced Pleural Lesions Exhibit Common Length-Dependent Molecular Changes (A) Left: transmission electron microscopy (TEM) images of the fiber panel; scale bars, 1 μm. Right: hematoxylin and eosin (H&E) images of the chest wall of mice at 1 and 12 weeks post-injection of the fiber panel (SFA, SNT, LFA, or LNT) compared to VC. Scale bars, 20 μm. (B) Cell types within the lesion were quantified (700–1,000 cells per cell marker, per treatment group; n = 4 per group) according to immunostaining (Figure S2) at 1 week and 12 weeks. (C) Gene expression pattern in control and fiber-exposed animals at 12 weeks post-injection. The heatmap displays the expression level of mRNAs extracted from the diaphragms of animals exposed to SFA, SNT, LFA, or LNT and VC (n = 4 per group). Legend bar shows the color code for the normalized intensity values. (D and E) Antibody array-based kinome profiling. (D) Common pattern of kinase activation induced by exposure to LFA and LNT at 1 and 12 weeks relative to VC (100%). (E) Activation of signaling pathways in human mesothelioma tissue from five patients is shown relative to kinase activity in normal primary mesothelial cells (control). (F) Human mesothelioma tissue was analyzed for phospho-STAT3 (Y705), phospho-ERK1/2 (T202/204), phospho-Akt (S473), and phospho-Src (Y418) by western blotting and compared to normal primary mesothelial cells (control). Representative data from six patients are shown. (G) Immunostaining of signaling proteins in paraffin-embedded sections of human mesothelioma tissue in relation to cancerous (M) and non-cancerous (L, lymphocytes; F, fibroblasts) cells. Representative data from three patients are shown. Scale bars, 100 μm. See also Figures S1–S3 and Tables S1–S3.

**Figure 2:**
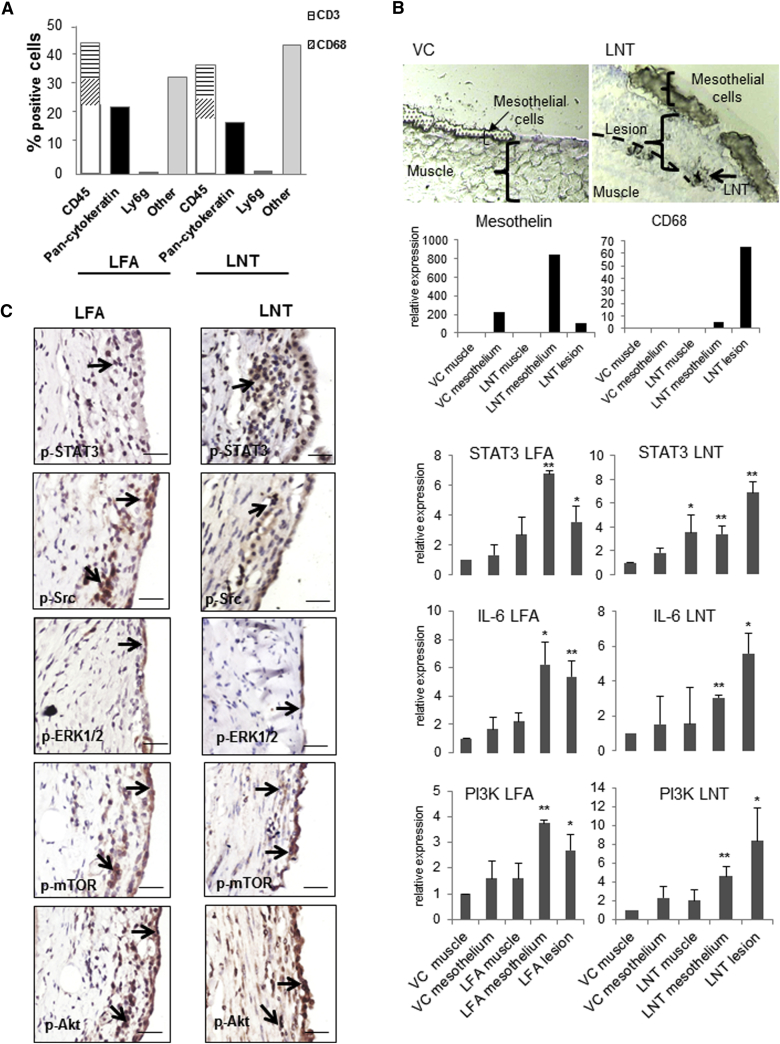
Sustained Inflammation and Activation of Pro-oncogenic Signaling Pathways in LFA- and LNT-Induced Lesions at 6 Months Post-injection (A) Cell types in LFA- and LNT-induced inflammatory lesions at 6 months post-injection were quantified by immunostaining with a panel of cell markers (pan-cytokeratin, mesothelial cells; CD68, macrophages; CD45, leukocytes; CD3, T cells; Ly6g, granulocytes). (B) Specific areas of tissue were isolated from fresh frozen diaphragms (n = 4) by laser microdissection in order to examine gene expression levels in different cell types (muscle, mesothelium, and lesion). For validation of the cell-type selection, the expression level of the cell markers mesothelin and CD68 in the different areas sampled was measured by qPCR. The expression level of the genes encoding STAT3, IL-6, and PI3K was examined by qPCR in muscle, mesothelium, and lesion (where present) microdissected from mice exposed to VC, LFA, or LNT at 12 weeks post-injection. ^∗^p < 0.05, ^∗∗^p < 0.01. (C) Activation of signaling pathways in the chest wall tissue of mice 6 months post-injection was analyzed by immunostaining. Positive staining for signaling proteins was observed in both mesothelial and non-mesothelial cells (black arrows). Scale bar, 20 μm. See also Figure S4.

Direct instillation of long, but not short, asbestos and CNTs into the pleural cavity of mice resulted in the development and marked progression of inflammatory lesions along the pleura (Figure 1A). The cellular profile of the lesions, comprising mesothelial cells and stromal cells as assessed by immunostaining, reflected the transition from acute to chronic inflammation [20] and was similar for both LFA and LNTs at 1 week, 12 weeks, and 6 months post-injection (Figures 1B and 2A).

mRNA array analysis (GEO: GSE51636) showed a common pattern of gene expression changes in both LFA- and LNT-induced lesions. When analyzed using hierarchical clustering, the samples from LFA- and LNT-exposed mice clustered together, whereas vehicle control (VC), SFA, and SNT groups formed a separate cluster (Figure 1C). The major network identified was associated with the inflammatory response (Table S1). To examine which signaling pathways were activated in CNT-exposed tissues, we determined the status of 64 kinases in pleurae from fiber-exposed and control animals. Antibody-based array analysis showed activation of pro-oncogenic signaling pathways, including Src family kinases, Akt, mTOR, ERK1/2, and STAT3, that was sustained in the pleurae of animals exposed to long, but not short, fibers (Figure S3). Acute and sustained kinase activation in both LFA- and LNT-exposed pleurae was strikingly similar, both in terms of the specific pathways identified and in their degree of activation (Figure 1D). Importantly, stimulation of these pathways was also observed in human mesothelioma tumor tissue from five patients compared to normal primary mesothelial cells (Figures 1E and 1F). Immunostaining of mesothelioma patient tissue demonstrated positive staining for the mesothelial marker HBME-1 and phosphorylated mTOR, 4EBP1, and Src in mesothelioma cells, as well as phospho-ERK1/2 in mesothelioma cells and fibroblasts, while phospho-STAT3 was predominant in lymphocytes and fibroblasts (Figure 1G).

Similar to that observed in mesothelioma patient samples, specific signaling pathways were activated in different cell types within LFA- and LNT-induced lesions. Thus, activation of ERK was restricted to the mesothelial cells overlaying the inflammatory lesions; Src, mTOR, and Akt were stimulated in both mesothelial cells and stroma, whereas STAT3 activation was most apparent in the stroma (Figure S4).

STAT3 expression is known to link inflammation and cancer [21]; therefore, to dissect the role of *Stat3* and related genes, we isolated mRNA from specific areas of the lesions by laser microdissection (Figure 2B), thus enabling quantification of gene expression to be correlated to different cell types (mesothelial cells and stroma). *Stat3* was upregulated (>3-fold) in both the mesothelial layer and stroma isolated from animals exposed to either LFA or LNTs, and its expression correlated with mRNA levels of *IL-6*, a well-characterized inducer of STAT3 in the IL-6/JAK2/STAT3 cascade [22, 23]. Signaling via the PI3K/mTOR axis is also important in asbestos-induced carcinogenesis [24]; consistent with this, the *Pik3cg* gene was upregulated in both LFA- and LNT-treated mice (Figure 2B).

### Progression of Fiber-Induced Lesions Is Characterized by Increased Proliferation and Oxidative DNA Damage

**Figure 3:**
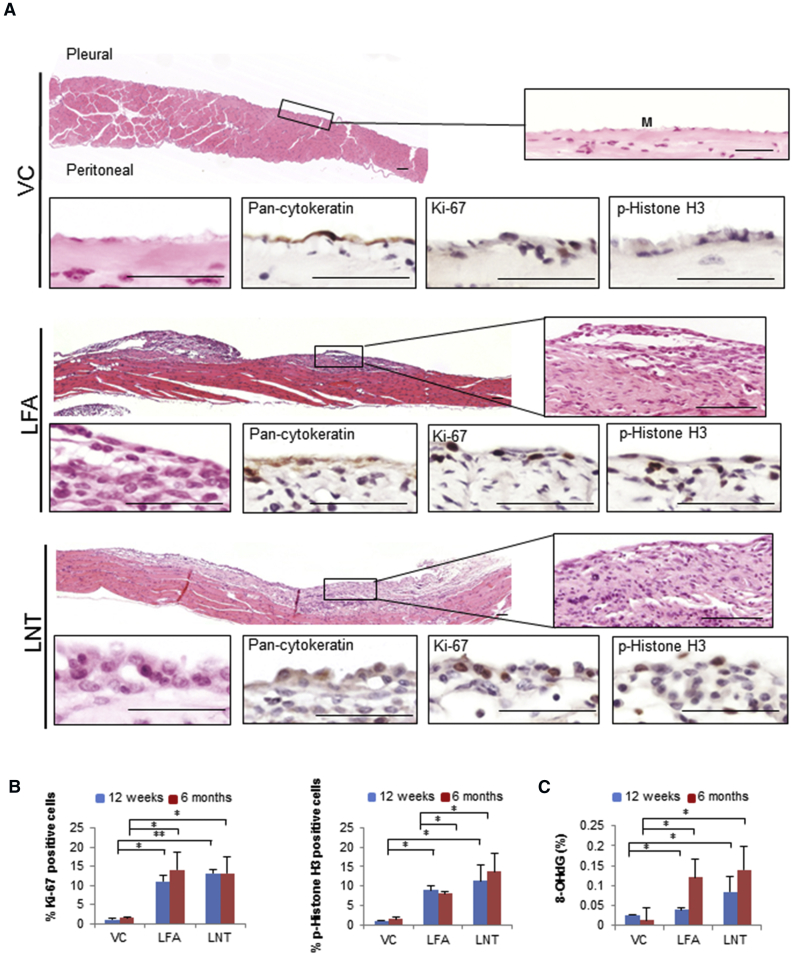
Progressing LNT-Induced Inflammatory Lesions Display Increased Proliferation and DNA Damage (A) Representative H&E-stained sections of pleurae from VC-, LFA-, and LNT-exposed mice at 6 months post-injection. Callouts show plump proliferating mesothelial cells (M) on the pleural surface of LFA- and LNT-exposed mice (positive for pan-cytokeratin; proliferation marker, Ki-67; and mitotic marker, p-Histone H3). Scale bars, 50 μm. (B) Increased proliferation in lesions of LFA- and LNT-exposed mice at 12 weeks and 6 months post-injection compared to VC, quantified by cells stained positively for Ki-67 and p-Histone H3 (700–1,000 cells per cell marker, per animal; n = 3 per group). (C) Sustained DNA damage in LFA- and LNT-induced lesions. The percentage of genomic DNA containing 8-hydroxy-2′-deoxyguanosine (8-OHdG) progressively increased in diaphragms of mice exposed to LFA or LNT compared to VC (n = 4 per group). Graphs (B and C) show mean ± SD; ^∗^p < 0.05, ^∗∗^p < 0.01 (two-tailed Student’s t test).

At 6 months post-injection, both LFA- and LNT-induced lesions expanded to become contiguous with the surface of the chest wall and diaphragm (Figure 3A), containing zones of dense fibrous stroma, active fibroblasts, and scattered mononuclear cells. Again, a distinct pattern of signaling pathway activation was common to both LFA- and LNT-induced lesions (Figure 2C). To assess the extent of proliferation resulting from sustained aberrant signaling, we examined expression of the proliferation marker Ki-67 and the mitotic marker p-Histone H3. Increased phosphorylation of Histone H3 is also associated with chromatin accessibility and forms part of an epigenetic regulatory mechanism required for malignant transformation [25, 26]. A sustained increase in proliferating cells was seen throughout both LFA- and LNT-induced lesions (Figure 3B). Oxidative DNA damage is often characteristic of chronic inflammation [27] and facilitates epigenetic modifications, thereby promoting carcinogenesis [28]. Importantly, the level of 8-hydroxy-2′-deoxyguanosine (8-OHdG), a marker of oxidative stress and genotoxicity, progressively increased to a similar degree in DNA samples from both LFA- and LNT-induced lesions (Figure 3C).

### LNTs and LFA Display Similar Carcinogenic Potential in the Pleura of Exposed Animals

**Figure 4:**
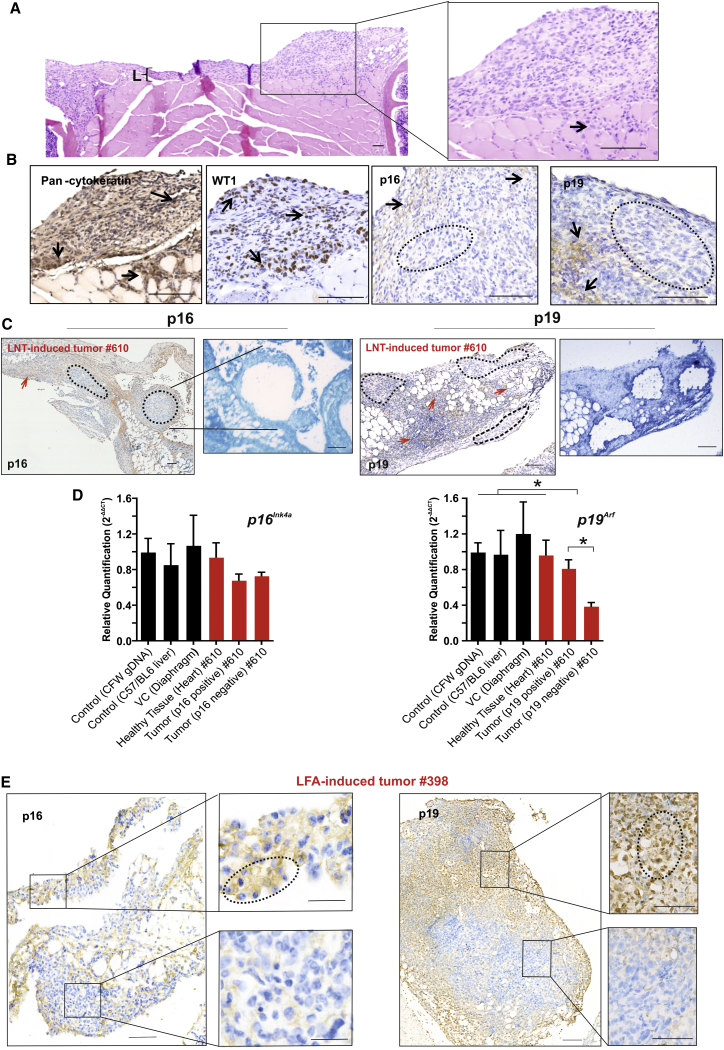
LNT- and LFA-Induced Inflammatory Lesions Progress to Malignant Mesothelioma with Disruption of *Cdkn2a* Gene and Encoded Proteins (A) LNT-induced mesothelioma at 12 months post-injection (animal ID: no. 610). The callout shows the mesothelioma composed of pleomorphic epithelioid tumor cells infiltrating into the underlying muscle (arrow). Adjacent to the tumor is an inflammatory lesion (L). Scale bars, 100 μm. (B) Immunostaining of LNT-induced mesothelioma (animal ID: no. 610). Tumor cells stained positively for the mesothelial cell markers pan-cytokeratin and WT1 (arrows); the tumor areas stained positively (arrows) or negatively (circle) for the *Cdkn2a*-encoded proteins p16 and p19. Scale bars, 100 μm. (C) Immunostaining of LNT-induced tumor (animal ID: no. 610) for p16 and p19. Negatively (circled) and positively (red arrows) stained areas were dissected and collected by power-assisted laser micro-dissection (PALM) for gDNA extraction and qPCR analysis. Callouts show a subsequent crystal violet-stained section of tumor after collection of selected areas. Scale bars, 100 μm. (D) Relative quantification (mean of 2^−ΔΔCT^) of *p16*^*Ink4a*^ and *p19*^*Arf*^ gene copy number in gDNA from micro-dissected tumor and healthy tissue from the same animal or VC, showing allelic loss of *p19*^*Arf*^ in p19-negative tumor areas compared to controls or p19-positive tumor areas. Graphs show mean ± SD; ^∗^p < 0.05 (significant difference is defined by *Z* score analysis; see also STAR Methods). (E) Immunostaining of LFA-induced tumor (animal ID: no. 398) for p16 and p19 protein. Callouts show positively stained areas (circled) in the upper panels and negatively stained areas in the lower panels that were dissected and collected by PALM for gDNA extraction and qPCR analysis (see Figure S6C). Scale bars, 100 μm. See also Figures S5–S7.

**Figure 5:**
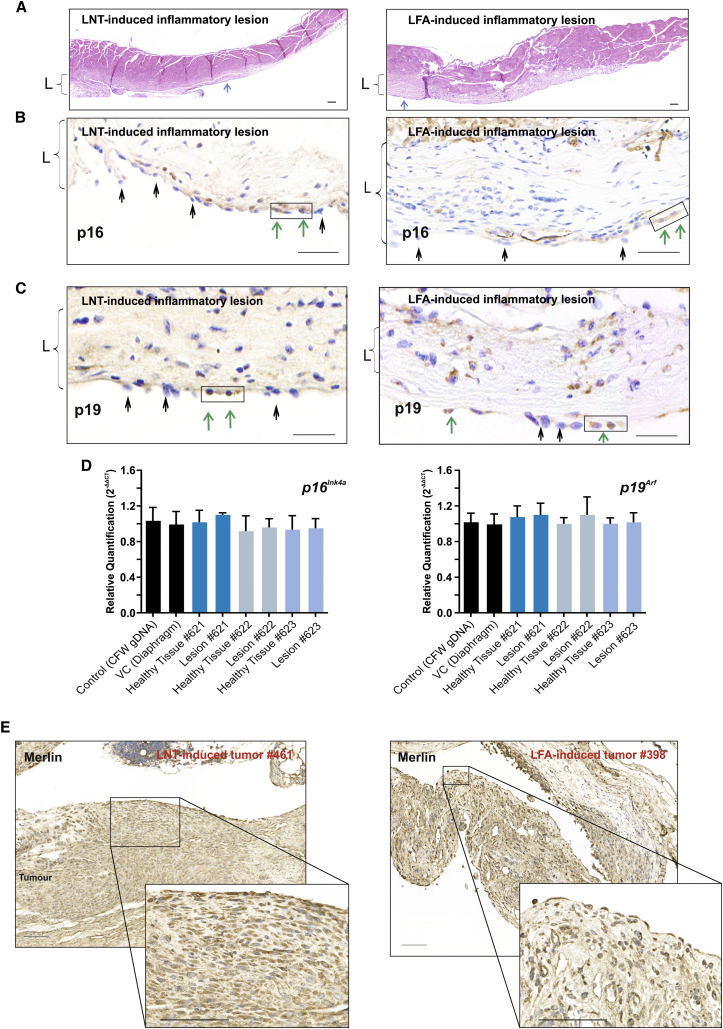
Chronic Inflammatory Lesions Induced by Long Fibers Display Loss of p16 and 19 Expression (A) Representative H&E-stained sections of diaphragm from LNT- and LFA-exposed animals that did not have tumors at the 1 year study endpoint, displaying extensive chronic inflammatory lesions (L) with fibrosis, infiltrating inflammatory cells, and plump reactive mesothelial cells on the surface of pleural lesions (blue arrows). Scale bars, 100 μm. (B) Loss of p16 expression in mesothelial cells in LNT- and LFA-induced inflammatory lesions. Representative images of immunostaining for p16 show predominantly negatively stained (black arrows) and only a few (boxed) positively stained (green arrows) mesothelial cells. Scale bars, 100 μm. (C) Loss of p19 protein expression in mesothelial cells in LNT- and LFA-induced inflammatory lesions. Representative images of immunostaining for p19 show predominantly negatively stained (black arrows) and only a few (boxed) positively stained (green arrows) mesothelial cells. Scale bars, 100 μm. (D) Relative quantification (mean of 2^−ΔΔCT^) of *p16*^*Ink4a*^ and *p19*^*Arf*^ gene copy number by qPCR analysis in gDNA isolated from positively and negatively stained mesothelial cells in the LNT-induced inflammatory lesions (animal IDs: nos. 621, 622, and 623) compared to controls. (E) Immunostaining for the *NF2*-encoded protein Merlin in LNT (animal ID: no. 461) and LFA (animal ID: no. 398) tumors. Representative images show positive cytoplasmic staining of tumor areas. Scale bars, 100 μm.

To explore the carcinogenic potential of long fibers in the pleura, we examined fiber-induced pathology at later time points (12–20 months) following exposure to LNTs or LFA. All fiber-exposed animals displayed advanced pleural lesions with mesothelial hyperplasia, fibrosis, and chronic inflammation (Figures 4A and 5A). In 10%–25% of animals across three independent studies, LNT-induced lesions progressed to pleural mesothelioma (1/4 wild-type animals exposed to 2.5 μg LNTs for 1 year; 1/4 animals exposed to 1 μg LNTs for 18 months, 1/5 animals exposed to 0.5 μg LNTs, and 1/12 animals exposed to 0.2 μg LNTs for 20 months) (Figures 4A, S5, and S7). Out of 32 animals exposed to asbestos (25 μg or 50 μg) for 18–20 months, three mice developed mesothelioma (Figure S6). This incidence of mesothelioma is consistent with the 15%–37% (overall or high levels of exposure, respectively) reported in humans exposed to asbestos [29].

Both LNT- and LFA-induced tumors, involving the chest wall, diaphragm, and pericardium, stained positive for the mesothelial markers pan-cytokeratin and WT1 and exhibited histopathology consistent with malignant mesothelioma [30] (Figures 4B, S6A, and S7A). Aberrant signaling pathway activation, observed in earlier lesions (Figures 2C and S4), was sustained in the LNT-induced tumors (Figure S5C).

To explore the molecular events underlying progression of LNT- and LFA-induced inflammatory lesions to mesothelioma, was examined the status of the tumor suppressor gene *Cdkn2a* and its products p16 and p19, known to be disrupted in asbestos-induced mesothelioma in humans [31, 32] (Figures 4B–4E, S6, and S7). Large areas of LNT-induced tumor (#610) or LFA-induced tumor (#398), which were predominantly negative for p16 or p19 protein expression, were micro-dissected and examined for *p16*^*Ink4a*^ and *p19*^*Arf*^ status (Figure 4C). In LNT-induced tumors, relative quantification of gene copy number [33] confirmed loss of the *p19*^*Arf*^ locus in the p19-negative areas, as evidenced by ∼60% reduction in *p19*^*Arf*^ genomic DNA (gDNA) compared to controls (Figure 4D). Loss of *p16*^*Ink4a*^, which is frequently co-deleted with *p19*^*Arf*^ [34], was not detected at this stage (Figures 4D), suggesting that allelic deletion of *p19*^*Arf*^ is an early event in LNT-induced carcinogenesis and indicating an alternative mechanism for p16 protein loss. Similarly, LNT tumor (#461) induced by a lower fiber dose (1 μg versus 2.5 μg) displayed loss of p16 and p19 protein expression, a reduction in *Cdkn2a* mRNA, and allelic loss of *p19*^*Arf*^ (Figure S7). In LFA-induced mesothelioma, loss of p16 and p19 protein was evident by a patchy pattern of immunostaining (Figure 4E) and reduced *Cdkn2a* mRNA levels (Figure S6B), although no reduction in *p19*^*Arf*^ gene copy number was detected at this stage (Figure S6C).

### Both LNTs and LFA Induce Disruption of Tumor Suppressor Genes in the Mesothelial Cells of Inflammatory Lesions

The molecular changes that occur during the latency period of mesothelioma and drive the transition from pre-neoplastic to neoplastic stage of disease are largely unknown. To explore whether disruption of *Cdkn2a* occurred prior to tumor development, we examined the status of *Cdkn2a* in laser-dissected mesothelial cells from advanced inflammatory lesions. LNT-induced chronic inflammatory lesions from animals that did not develop tumors at the 1 year study end point (Figure 5A) displayed no reduction in *p16*^*Ink4a*^ or *p19*^*Arf*^ gene copy number (Figure 5D); however, mRNA levels were reduced (data not shown), and both p16 and p19 protein expression was absent in the majority of mesothelial cells in LNT-induced lesions (Figures 5B and 5C). Consistent with loss of the *CDKN2A*-encoded proteins p16/p14 in human mesothelioma, loss of p16 and p19 protein expression was also evident in advanced LFA-induced lesions from animals that did not develop mesothelioma at the 1 year study end point (Figures 5B and 5C). No loss of *NF2*-encoded Merlin expression was detected in either LFA- or LNT-induced tumors (Figure 5E). Mesothelial expression of Merlin in the LFA- and LNT-induced inflammatory lesions was not altered (data not shown).

**Figure 6:**
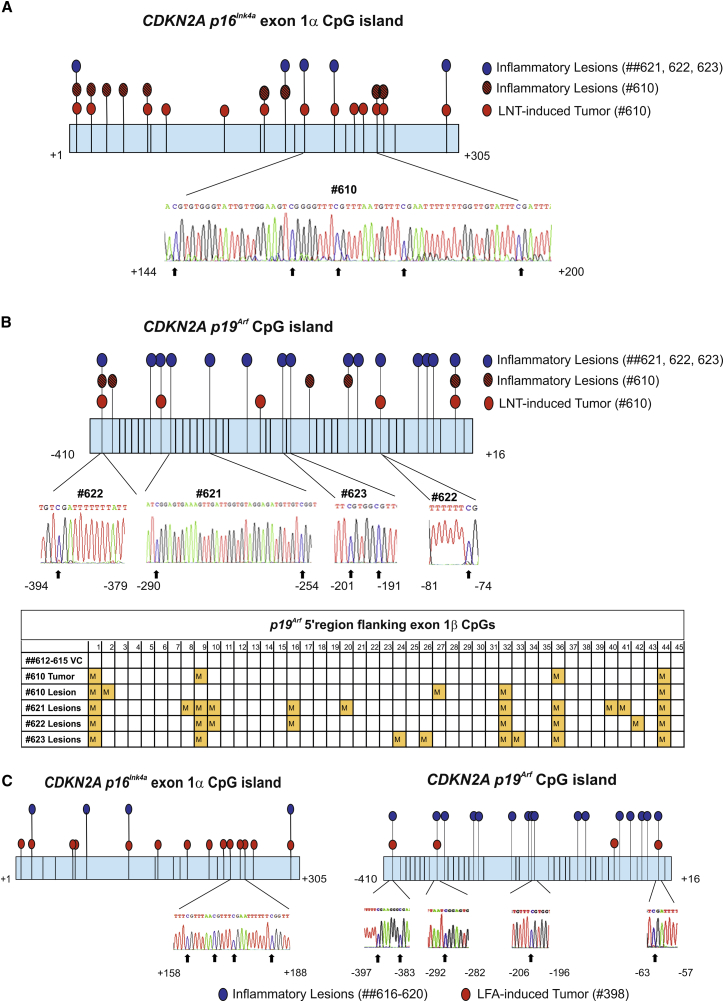
Chronic Inflammatory Lesions and Malignant Mesothelioma Induced by LFA and LNT Display Hypermethylation of the *Cdkn2a* (*p16* ^*Ink4a*^*/p19*^*Arf*^) Locus (A) Schematic representation of the hypermethylation profile of the *p16*^*Ink4a*^ CpG island in exon 1α determined by bisulphite sequencing of gDNA. All CpG dinucleotides are shown by vertical lines. Hypermethylated CpG sites were identified in gDNA extracted from p16-negative areas of LNT-induced tumor (animal ID: no. 610; red-filled circles), in gDNA isolated from mesothelial cells in inflammatory non-neoplastic lesions from the same animal (hatched circles), and in gDNA from mesothelial cells in chronic inflammatory lesions of LNT-exposed animals that did not develop tumors at the 1 year study endpoint (animal IDs: nos. 621, 622, ad 623; blue-filled circles). The call-out shows a region of bisulphite converted sequence with unconverted cytosines (arrows). (B) Schematic representation of the hypermethylation status of the 5′ *p19*^*Arf*^ CpG island located upstream of exon 1β determined by bisulphite sequencing. All CpG dinucleotide positions are shown by vertical lines. Hypermethylated CpG sites were identified in p19-negative areas of LNT-induced tumor (no. 610; red-filled circles), in mesothelial cells isolated from inflammatory non-neoplastic lesions of the same animal (hatched circles), and in mesothelial cells isolated from chronic inflammatory lesions of LNT-exposed animals that did not develop tumors at the 1 year study endpoint (animal IDs: nos. 621, 622, and 623; blue-filled circles). Callouts show examples of unconverted cytosines in hypermethylated CpGs. Results are summarized in the table, where each yellow-filled square represents multiple sequencing runs (unconverted CpG detected in 10–60 clones compared to none in VC). (C) Schematic representation of the hypermethylation profile of the *p16*^*Ink4a*^ CpG island in exon 1α and the 5′ *p19*^*Arf*^ CpG island located upstream of exon 1β determined by bisulphite sequencing. Hypermethylated CpG sites were identified in gDNA extracted from p16-negative areas of LFA-induced tumor (animal ID: no. 398; red-filled circles) and in gDNA from mesothelial cells in chronic inflammatory lesions of LFA-exposed animals that did not develop tumors at the 1 year study endpoint (animal IDs: nos. 616–620; blue-filled circles). The callout shows a region of bisulphite converted sequence with unconverted cytosines. See also Figure S7.

DNA hypermethylation is frequently associated with tumor suppressor gene transcriptional inactivation. Importantly, loss of function often occurs during the early stages of carcinogenesis and predominately at the pre-cancerous stage [7, 8]. To explore possible epigenetic mechanisms of fiber-induced carcinogenesis, we examined gDNA from animals with advanced LNT-induced inflammatory lesions and inflammatory lesions and/or tumors from animals with LNT-induced mesothelioma (#610 and #461), as well as inflammatory lesions and/or tumors from animals with LFA-induced mesothelioma (#398), for *p16*^*Ink4a*^ or *p19*^*Arf*^ methylation status. Bisulphite sequencing confirmed hypermethylation of CpG islands in *p16*^*Ink4a*^ and *p19*^*Arf*^ (located in exon 1α and the 5′ region flanking exon 1β, respectively) in mesothelial cells in advanced LNT- and LFA-induced lesions, as well as in LFA- and LNT-induced tumors, compared with VC (Figures 6 and S7E).

## Discussion

**Figure 7:**
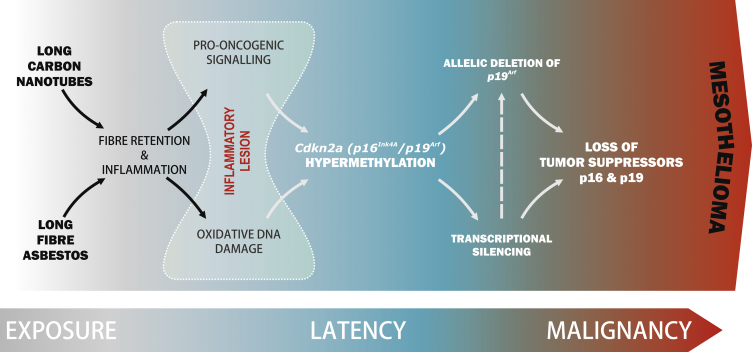
LNTs Replicate Asbestos-Induced Mesothelioma with Disruption of the Tumor Suppressor Gene *Cdkn2a* (*Ink4a/Arf*) Schematic depicting the sequence of events in the pleurae of animals exposed to LFA or LNT showing commonality in LFA- and LNT-induced disease progression that replicates mesothelioma development in humans.

Here, we show for the first time that common molecular events underlie the development of LFA- and LNT-induced pleural lesions that progress to mesothelioma (Figure 7). Aberrant cell signaling was detected as early as 1 week post-instillation and sustained during the entire time of disease progression, including end-stage disease, in mice. Importantly, this pattern of pro-oncogenic signaling was strikingly similar in mesothelioma patients. The potential role of the stromal component of human mesothelioma in tumor progression has been highlighted previously [35, 36]. In this regard, we now show that sustained activation of pro-oncogenic signaling pathways in fiber-induced lesions occurs largely in non-mesothelial (stromal) cells as fiber-induced lesions progress. Our data therefore suggest that proliferation of the mesothelial cell layer and the presence of “reactive” (changed morphology) mesothelial cells within the lesions occurs as a consequence of the crosstalk between stromal cells with activated pro-oncogenic pathways and target mesothelial cells.

Inflammatory signals cause alterations in the cellular epigenetic program and induce hypermethylation [37]. The presence of oxidative DNA damage could also contribute to pro-oncogenic events within the microenvironment, favoring aberrant DNA methylation in target cells [38], with hypermethylation of *Cdkn2a* (*Ink4a/Arf*) in LNT- and LFA-induced lesions prior to tumor development leading to allelic loss of *p19*^*Arf*^, as has been suggested in other cancers [17]. Consistent with a role for hypermethylation, DNMT3a and DNMT3b mRNA levels were increased in mesothelial cells from LFA- and LNT-induced tumors (data not shown).

Alteration of the *CDKN2A* locus in human malignant mesothelioma has been reported with inactivation of p16 in more than a half of mesothelioma patients [9] and, in more recent studies, with hypermethylation and silencing of p19 in 44% of patients [39].

Significantly, we discovered that epigenetic silencing of *Cdkn2a* (*Ink4a/Arf*) and deletion of *p19*^*Arf*^ observed in LNT-induced tumors recapitulates common features of human asbestos-induced mesothelioma (Figure 7). Overall, these findings provide important new insights into the early molecular changes that occur during the long latency period between fiber exposure and mesothelioma development and identifies epigenetic and/or genetic disruption of *Cdkn2a* as a key event in long-fiber-induced malignant transformation (Figure 7). Importantly, these findings facilitate the identification of potential biomarkers for earlier detection of asbestos-induced mesothelioma, as well as the development of new therapeutic avenues by which to tackle early-stage disease.

Immunostaining of both LFA-and LNT-induced mesotheliomas for the tumor suppressor protein BAP1 revealed positive cytoplasmic staining in all tumor cells and positive nuclear staining in ∼10% tumor cells (data not shown). In view of the latest findings of separate activities of BAP1 in the nucleus and cytoplasm, as well as a possible requirement for both cytoplasmic and nuclear forms to exert a tumor suppressor function, the molecular involvement of BAP1 in carbon nanofiber-induced carinogenesis will be a focus of our future work.

The long latency of malignant mesothelioma, with decades-long chronic inflammation accompanied by an aberrant microenvironment and the presence of ROS and oxidative DNA damage, would advocate a multifactorial mechanism of disease development, with a clear contribution via loss of tumor suppressor genes such as p16 and p19, as reported here.

Overall, the common signature of LFA- and LNT-induced pathology demonstrates that there is a conserved molecular mechanism through which long fibers induce pleural disease, including mesothelioma, and crucially our data place long CNT fibers on the same adverse outcome pathway as asbestos. Notably, other nanofibers have been shown to produce length-dependent inflammatory effects in the pleura similar to LNTs [40], suggesting that any respirable long fiber that is biopersistent may pose a similar hazard. Given that the increasing manufacture of long CNT fibers raises the potential for human exposure, our findings reinforce the need for caution when using these agents if long-term harm is to be avoided.

## STAR★Methods

### Key Resources Table

**Table:** 

REAGENT or RESOURCE	SOURCE	IDENTIFIER
**Antibodies**		

Rabbit monoclonal anti- phospho-Histone H3 (Ser10) (clone D2C8) XP	Cell Signaling Technology	Cat #3377S; RRID: AB_1549592
Rabbit polyclonal anti-Ki-67	Abcam	Cat# ab15580; RRID: AB_443209)
Rabbit monoclonal anti-Stat3 (79D7) mAb antibody	Cell Signaling Technology	Cat# 4904; RRID: AB_331269
Rabbit monoclonal anti- phospho-STAT3 (Tyr705 / Ser727) unconjugated, (clone EP2147Y)	Abcam	Cat# ab76315; RRID: AB_1658549
Rabbit monoclonal anti- phospho-Akt (Ser473) (clone 193H12)	Cell Signaling Technology	Cat# 4058; RRID: AB_331168
Rabbit polyclonal anti- Akt	Cell Signaling Technology	Cat# 9272; RRID: AB_329827
Rabbit anti- p44/42 MAP kinase (phosphorylated Erk1/2)	Cell Signaling Technology	Cat# 9101; RRID: AB_331646
Rabbit polyclonal anti-p44/42 MAPK (Erk1/2)	Cell Signaling Technology	Cat# 9102; RRID: AB_330744
Rabbit polyclonal anti-phospho-Src Family (Tyr416)	Cell Signaling Technology	Cat# 2101; RRID: AB_331697
Rabbit monoclonal anti-Src (clone 36D10)	Cell Signaling Technology	Cat# 2109; RRID: AB_2106059
Mouse monoclonal anti-beta-Actin, unconjugated, (clone AC-15)	Sigma-Aldrich	Cat# A1978; RRID: AB_476692
Rabbit polyclonal anti-CD45	Abcam	Cat# ab10558; RRID: AB_442810
Rabbit polyclonal anti-CD68	Abcam	Cat# ab125212; RRID: AB_10975465
Rat monoclonal anti-CD3	Abcam	Cat# ab56313; RRID: AB_940876
Rabbit polyclonal anti-NF2 (clone A-19)	Santa Cruz Biotechnology	Cat# sc-331; RRID: AB_2298548
Rabbit polyclonal anti-p16 (clone M-156)	Santa Cruz Biotechnology	Cat# sc-1207; RRID: AB_632106
Rabbit polyclonal anti-CDKN2A/p19ARF	Abcam	Cat# ab80; RRID: AB_306197
Rabbit polyclonal anti-phospho Src (Y418)	Abcam	Cat# ab47411; RRID: AB_870740
Rabbit polyclonal anti-phospho mTOR (S2448)	Abcam	Cat# ab51044; RRID: AB_2247119
Rabbit polyclonal anti-phospho AKT1 (Ser473)	Abcam	Cat# ab6613; RRID: AB_1140998
Rabbit monoclonal anti-phospho STAT3 (Y705) (clone EP2147Y)	Abcam	Cat# ab76315; RRID: AB_1658549
Mouse monoclonal anti- HBME-1	Abcam	Cat# ab2383; RRID: AB_303026
Rat monoclonal anti- Ly6g (cloneRB6-8C5)	Abcam	Cat# ab25377; RRID: AB_470492
Rabbit monoclonal anti-phospho-p44/42 MAPK (Erk1/2) (Thr202/Tyr204) (clone 197G2)	Cell Signaling Technology	Cat# 4377; RRID: AB_331775
Rabbit polyclonal pan-Cytokeratin (clone H-240)	Santa Cruz Biotechnology	Cat# sc-15367; RRID: AB_2134438
Mouse monoclonal anti-Wilms Tumor Protein 1 (clone 6F-H2)	Dako	Cat# M3561, RRID: AB_2304486

**Biological Samples**		

gDNA from Swiss-Webster albino (CFW) mice	Promega	Cat#G3091
Patient-derived mesothelioma tissue	This paper	N/A

**Chemicals, Peptides, and Recombinant Proteins**		

Long Carbon Nanotubes	University of Manchester, (Manchester, UK)	Produced and characterized by Dr. Ian Kinloch
South African amosite	Manville Corporation, USA	N/A
Short Carbon Nanotubes	Nanostructured & Amorphous Materials (TX, USA)	Cat#1246YJS
TRIzol	Fisher Scientific	Cat#12044977
Invitrogen SuperScript III Reverse Transcriptase	Fisher Scientific	Cat#18080093

**Critical Commercial Assays**		

Phospho-Kinase Array Kit	R&D Systems	Cat#ARY003b
SurePrint G3 Mouse GE 8x60K Microarray Kit	Agilent Technologies	Cat#G4852B
Agilent Low Input Quick Amp one-color Labeling Kit	Agilent Technologies	Cat#5190-2305
PureLink Genomic DNA Mini Kit	Fisher Scientific	Cat#K182001
GeneJET Genomic DNA Purification Kit	Fisher Scientific	Cat#K0721
EpiQuik 8-OHdG DNA Damage Quantification Direct Kit (Colorimetric)	Insight Biotechnology	Cat#P-6003-48
EZ DNA Methylation-Direct Kit	Cambridge Bioscience	Cat#D5020
TOPO TA Cloning Kit for Sequencing, with One Shot TOP10 Chemically Competent *E. coli*	Fisher Scientific	Cat#K457501
Histostain Plus Broad Spectrum	Invitrogen	Cat#859043
LSAB2 System-HRP	Dako	Cat#K0675
Rabbit specific HRP/DAB (ABC) Detection IHC Kit	Abcam	Cat#ab64261
TaqMan Genotyping Master Mix	Fisher Scientific	Cat#4371355
SYBR Green PCR Master Mix	Applied Biosystems	Cat#4309155

**Deposited Data**		

The “Gene changes in response to asbestos and carbon nanotube exposure in the pleural cavity, measured using microarrays” have been deposited in the NCBI GEO database https://ww.ncbi.nlm.nih.gov/geo/ under ID code GSE51636	https://www.ncbi.nlm.nih.gov/geo/query/acc.cgi?acc=GSE51636	GEO: GSE51636

**Experimental Models: Cell Lines**		

Human adult mesothelial cells, 4 female donors	Zenbio	Cat#MES-F

**Experimental Models: Organisms/Strains**		

Mouse: C57BL/6	Charles River Laboratories	Strain code 027

**Oligonucleotides**		

Primers for qPCR, Gene copy number and Bisulfate Sequencing, see Table S4	This paper	N/A

**Software and Algorithms**		

Ingenuity Pathways Analysis software	Ingenuity Systems	Cat#830003
Primer Express v3.0.1 Software	Fisher Scientific	Cat#4363991
GeneSpring GX Software	Agilent	Cat#G3778AA

### Contact for Reagent and Resource Sharing

Further information and requests should be directed to and will be fulfilled by the Lead Contact, Marion MacFarlane (mm21@le.ac.uk).

### Experimental Model and Subject Details

#### Experimental animals

Eight-week-old female C57BL/6 strain mice (Charles River Laboratories, UK) were used in this study. Mice were kept in a maximal group size of five in standard caging with sawdust bedding within a pathogen-free Home Office approved facility. Mice were maintained on a normal 12 hr light and dark cycle. Prior to treatment, mice were kept for 7 days in the facility to acclimatize. The work was carried out by staff holding a valid UK Home Office personal license under a Home Office approved project license.

#### Human subjects

Informed consent was obtained from all subjects with Ethical Committee Approval – LREC 08/H0406/226. Tissues from 13 patients were analyzed in this study, including 1 female and 12 males aged 45–78 years [41].

#### Fiber samples

The fiber panel consisted of one sample of short, straight CNT (SNT), one sample of long, straight CNT (LNT), short-fiber amosite asbestos (SFA) and long-fiber amosite asbestos (LFA). The SNT sample, produced by the catalytic vapor discharge method (CVD), was purchased commercially (Nanostructured & Amorphous Materials, TX, USA). The LNT sample was produced in an academic research laboratory (University of Manchester, Manchester, UK) using the CVD method. Mixed-length amosite asbestos enriched for long fibers (LFA), and shortened amosite asbestos [17] were used to link the response to asbestos pathogenicity. Both LFA and SFA were created from the same batch of South African amosite [18] obtained from the Manville Corporation, United States. SFA was prepared by grinding long fibers in a ceramic ball mill, and the resulting fiber preparation sedimented in water. Physical characteristics (diameter and length) were measured from scanning electron microscopy (SEM) and transmission electron microscopy (TEM) images of the dispersed fiber samples, as previously described by Poland et al. [11] (Figure S1). Trace soluble metal contaminants previously tested and reported by Poland et al. [11] were low and, thus, not considered to play a role in these studies.

#### Intrapleural injection

Injection directly into the pleural space without perforating the lungs was enabled by the addition of a sleeve over the tip of the 27-Gauge needle, which prevented the needle from passing through the pleural space into the lungs [1]. Mice were randomly allocated to the treatments and received injection of one of the following: vehicle control (VC), SFA, LFA, SNT or LNT. For 1 and 12 weeks exposure, CNT samples were injected into the pleural cavity at a dose of 5 μg/mouse, n = 4 in each treatment group. For 6 months and 1year exposure, the treatments were as follows: VC, LFA and LNT, with n = 4 in each treatment group, at a dose of 2.5 μg/mouse (100 μL) of LNT. For prolonged (up to 20 months) exposure, mice were injected with 1 μg/mouse of LNT (n = 4), 0.5 μg/mouse of LNT (n = 5) or 0.2 μg/mouse of LNT (n = 12). Asbestos fiber samples were administered at a dose of 25 μg/mouse (100 μL) (n = 4 for short-term studies and n = 16, prolonged exposure) or at 50 μg/mouse (n = 16, prolonged exposure). 0.5% BSA/saline (100 μL) was injected into mice as a vehicle control (VC). After 1 week, 12 weeks, 6 months, 1 year and 18-20 months, mice were humanely killed and tissues were collected for further examination. Animal IDs, indicated in the Figures, were as follows: #610 - LNT-exposed mouse that developed mesothelioma at 1 year study completion point; ## 621, 622, 623 - LNT-exposed mice that did not have a tumor at 1 year study completion point; ## 612, 613, 614, 615 - VC; #398 - LFA-exposed mouse; #461 - low-dose LNT-exposed mouse that developed mesothelioma after 17 months exposure; ## 616-620 - LFA-exposed mice with inflammatory lesions.

### Method Details

#### Experimental Design

All experiments were reproduced in at least three biological repeats. For *in vivo* experiments, sample sizes were chosen on the basis of prior experiments which have elicited significant results with a similar number of mice. With the exception of histological analysis, data collection and analyses were not performed blind. Data were considered statistically significantly different at p < 0.05. No data were excluded from any dataset.

#### Tissue dissection

The lower-right posterior portion of the chest wall and half of the diaphragm were carefully removed from the mice. The tissue was washed in ice-cold saline, fixed in 10% formalin for 4 hr and transferred to 70% ethanol. Samples were embedded in paraffin, sectioned, and used for H&E, immunostaining and PALM microdissection. The pleurae were dissected from the surface of the chest wall and snap frozen for kinome profiling. The other half of the diaphragm was snap frozen for RNA extraction and PALM microdissection.

#### Kinome profiling

Kinome profiling was performed using Phospho-Kinase Array Kit according to manufacturer’s protocol (R&D Systems, Oxford, UK). Pleural tissues from fiber-exposed and control mice were collected by dissection, homogenized and lysed, the lysates from the pleurae of 4 animals were pooled for each treatment group. Lysates, cleared by centrifugation, were loaded onto the provided membranes pre-coated with capture antibodies, and the presence of bound phospho-proteins was determined by western blotting with a mixture of detection antibodies. The signal intensities for kinase phosphorylation were determined in duplicate by densitometry and normalized on the provided positive control. The status of kinase phosphorylation in the pleurae of fiber-exposed animals is reported relative to the vehicle control.

#### RNA microarrays

Total RNA from control and fiber-exposed animals (n = 4) was extracted by TRIzol (Fisher Scientific, Loughborough, UK) and then used for labeling and hybridization. Hybridization to 60K whole mouse genome microarray gene expression chips was conducted following manufacturer’s protocol (Agilent Technologies, Berkshire, UK). Briefly, total RNA from control and fiber-exposed mice, 12 weeks post-injection, was used for labeling and hybridization. RNA samples were Cy3-labeled using Agilent Low Input Quick Amp 1-color Labeling Kit (Agilent Technologies, Berkshire, UK). The level of dye incorporation was evaluated using a spectrophotometer (Nanodrop ND1000, LabTech). Labeled RNA was then fragmented in the appropriate buffer (Agilent Technologies, Berkshire, UK) for 30 min at 60°C before dilution (v/v) in hybridization buffer. Hybridization to 60K high-density oligonucleotide microarray slides was performed in a microarray hybridization oven (Agilent Technologies, Berkshire, UK) overnight at 65°C. Following hybridization, the slides were rinsed in gene expression wash buffers 1 and 2 and immediately scanned using a DNA Microarray Scanner (Model G2505C, Agilent Technologies, Berkshire, UK).

#### Immunohistochemistry

Chest wall or diaphragm sections (5 μm) were deparaffinized with xylene and rehydrated. Antigen retrieval was carried out by incubating the slides in citrate buffer (pH 6.0) at 95°C for 15 min. Sections were blocked with 10% goat serum for 30 min at room temperature. Primary antibodies were diluted in 1% goat serum/0.1% BSA/PBS. Sections were incubated with primary antibodies (KEY RESOURCE TABLE) overnight at 4°C. Sections were washed with Tris-buffered saline with 0.1% Tween 20 (TBST) and incubated with 3% H_2_O_2_ for 10 min at room temperature to block endogenous peroxidase activity. Sections were washed before incubation with biotin-labeled secondary antibodies (anti-Rabbit: Abcam, pre-diluted; anti-Mouse: DAKO LSAB2 System-HRP, anti-Rat: Invitrogen Histostain Kit pre-diluted) for 30 min at room temperature. Staining was visualized using a HRP/DAB detection system Abcam (Cambridge, UK) or Histostain-Plus detection System (Life Technologies). Control IHC experiments (data not shown) were performed without primary antibody. All sections were counterstained with Gill’s hematoxylin and mounted for digital slide scanning using a Hamamatsu slide scanner (NanoZoomer-XR Digital slide scanner C12000-01; Welwyn Garden City, Hertfordshire, UK). An ImageJ Color Balance Plugin, set with the same scaling factor for all images, was applied to all scanned images to normalize scanner background.

The proportion of each cell type in the lesions was determined by counting the number of positively stained cells in 5 random fields of view and dividing by the total number of cells (between 700-1000 cells per cell-marker per treatment group were counted).

Quantification of Ki-67 and phospho-Histone H3-positive cells was carried out by counting the number of positively stained cells in 5 random fields of view and dividing by the total number of cells (between 700-1000 cells per animal were counted, n = 3 per group). Data presented as mean ± SD. Statistical significance of data was estimated using two-tailed Student’s t test.

#### Laser microdissection and RNA analysis

Collection of tissues for RNA from selected areas of normal diaphragms (VC) and fiber-induced lesions (LFA, LNT) and collection of tissues for genomic DNA (gDNA) from tumor areas, predominantly positively or negatively stained for p19 or p16 protein, and from control tissues was performed by contact-free laser microdissection using the PALM Robot-MicroBeam system (P.A.L.M. Microlaser Technologies AG, Bernried, Germany), Motorised Zeiss Observer Z.1 inverted microscope (Zeiss, Germany) with high precision XY stage, pulsed UV laser and Robomover (PALM RoboSoftware, P.A.L.M. Microlaser Technologies AG, Germany). Laser catapulting was used for non-contact capture of excised samples in microcentrifuge tubes. Total RNA was isolated by using TRIzol (Fisher Scientific, Loughborough, UK). First strand cDNA synthesis was carried out using Superscript III (Fisher Scientific, Loughborough, UK). PCR primers were selected using the Primer Express v3.0.1 Software program (Fisher Scientific, Loughborough, UK). Primer sequences are shown in Table S4. Primers were designed to cross exon-exon boundaries and the concentration optimized (300 - 900 nM) to ensure that the efficiency of the target gene amplification and the efficiency of the endogenous reference amplification are approximately equal. Real-time PCR was performed using SYBR Green PCR Master Mix, primers, and 10 ng of reverse-transcribed cDNA in the ABI PRISM 7500 Sequence Detection System (Applied Biosystems, Foster City, CA). The thermal-cycler protocol was: stage 1, 50°C for 2 min; stage 2, 95°C for 10 min; and stage 3, 40 cycles at 95°C for 15 s and 60°C for 1 min. Each sample was run in triplicate. The C_T_ values for the target amplicon and endogenous control β2-microglobulin were determined for each sample. Quantification was performed using the comparative C_T_ method ΔΔC_T_). Data presented as mean ± SD (n = 4). Statistical significance was assessed as p < 0.05 using two-tailed Student’s t test.

#### Immunoblotting

Proteins were extracted from snap frozen freshly-resected mesothelioma tumor samples using lysis buffer (0.5% NP-40, 20 mM Tris-HCl (pH 8.0), 137 mM NaCl, 10% glycerol, 2 mM EDTA, 1 mM sodium orthovanadate, 10 μg/μL leupeptin, and 10 μg/μL aprotinin) and brief sonication and run on SDS-PAGE (20 μg protein). Proteins were transferred onto nitrocellulose membranes (Bio-Rad laboratories, Hemel Hempstead, UK) using electrophoresis. Membranes were pre-incubated with 5% skimmed milk in TBS-T. After incubation with primary and secondary antibodies, bands were detected by enhanced chemiluminescence (GE Healthcare, Little Chalfont, Buckinghamshire, UK) and visualized by exposure to X-ray films (Hyperfilm ECL; Amersham Biosciences, Chalfont St Giles, UK). Primary antibodies were from the following sources:anti-STAT3, anti-phospho-Akt (Ser 473), anti-Akt, anti-phospho-p44/42 MAPK (ERK1/2) (Thr202/Tyr204), anti-p44/42 MAPK (ERK1/2), anti-phospho Src (Y418), anti-Src from Cell Signaling Technology, (Buckinghamshire, UK); rabbit anti-phospho-Stat3 (Tyr705) from Abcam (Cambridge, UK), mouse anti-β Actin (loading control) was from Sigma–Aldrich (Gillingham, UK).

#### DNA damage assessment

Genomic DNA was isolated from the diaphragms of mice exposed to LFA and LNT for 12 weeks or 6 months using a DNA isolation kit GeneJET Genomic DNA Purification Kit (Thermo Fisher Scientific, Paisley, UK); 8-OHdG was measured using an EpiQuik 8-OHdG DNA Damage Quantification Direct Kit (Colorimetric) (Insight Biotechnology Ltd, Wembley, UK) according to manufacturer instructions and compared to VC (n = 4 per group). Briefly, 300 ng genomic DNA was bound to strip wells with high DNA affinity and 8-OHdG was detected using capture and detection antibodies. The detected signal was enhanced and then quantified colorimetrically in a microplate reader. Quantification was performed by using a standard curve and plotting the OD values versus the amount of positive control at each concentration point. Data presented as mean ± SD. Statistical significance of the data was estimated using a two-tailed Student’s t test.

#### Relative quantification of gene copy number by real-time PCR

An assay based on the paralog ratio test and real-time PCR was used to determine the DNA copy number in formalin fixed paraffin embedded (FFPE) tissue as described previously [33]. Genomic DNA (gDNA) was extracted using PureLink Genomic DNA Mini Kit (Fisher Scientific, Loughborough, UK) accordingly to the manufacturer’s instruction, from tumor areas positively and negatively stained for p16 and p19, and control tissues including unaffected tissue (heart) from fiber-exposed mice, diaphragm tissue from VC group, and liver tissue from a C57/Bl6 mouse. Commercially available murine gDNA from Swiss-Webster albino (CFW) mice (Promega, Southampton, UK) was also included as a control. Briefly, PCRs were designed in which two target sequences were amplified by a pair of primers. The first gene in each pair was from a region of a frequent loss or deletion (p16 or p19), whereas the second gene was the “housekeeping” gene, Supervillin, from a different chromosome. The Primer Express v3.0.1 Software program (Fisher Scientific, Loughborough, UK), was used to identify primer pairs and to design minor groove-binding hydrolysis probes. Primer sequences (Sigma) and TaqMan Probes (Life Technologies) are listed in Table S4.

Real-time PCRs were performed on a 96-well plate on the ABI 7500 Fast system (Applied Biosystems Foster City, CA). Each of 10 μL reactions contained 3.4 μL of gDNA (5 ng), 0.3 μL of each of the two primers required for each locus (20 pmol/L), 0.2 μL of each of the probes required (50 to 200 nmol/L), and 5 μL of Genotyping MasterMix (Applied Biosystems Foster City, CA). Master mixes were prepared for each reaction, and the master mixes and DNA samples were transferred to 96-well plates using an electronic multi-channel pipette. The PCR conditions were as follows: 2 min at 50°C; 10 min at 95°C; 15 s at 95°C and 60 s at 60°C. Fifty cycles were performed. C_T_ values were determined using the automatic threshold settings. Four samples of gDNA (unaffected heart tissue from the same animal, diaphragm tissue from a VC mouse, liver from C57/BL6 mouse, and commercial gDNA from Swiss-Webster albino (CFW) mice (Promega, Southampton, UK) were used as controls.

#### Bisulphite sequencing

Genomic DNA from selected areas of tumor or from mesothelial cells in the inflammatory lesions was isolated using GeneJET Genomic DNA Purification Kit (Fisher Scientific, Loughborough, UK) and was bisulphite converted and recovered with EZ DNA Methylation-Direct Kit (Cambridge Bioscience, Cambridge UK), as recommended by manufacturer. The PCR to amplify CpG islands in the genes of interest was performed on a ^3^Prime Thermal Cycler (Bibby Scientific Limited, Stone, UK) using EpiMark Hot Start *Taq* DNA Polymerase (New England Biolabs Ltd, Herts, UK). The PCR conditions were as follows: 95°C for 3 min, then 40 cycles of 95°C for 30 s, 55°C for 30 s and 67°C for 2 min. A final incubation at 67°C for 10 min concluded the PCR. Positive control (Universal Methylated Mouse DNA Standard, Millipore Ltd, Watford, UK) and control (normal mesothelial tissue from VC) samples were included in each PCR reaction. PCR products were verified by gel electrophoresis, and a small aliquot of the PCR reaction was used with the TOPO-TA cloning system (*Thermo Scientific,* Paisley, UK) as suggested by the manufacturer. Clones were picked from Lysogeny Broth-Ampicillin cultures, and were then screened using restriction analysis. Positive clones were sequenced using M13 primer by the University of Leicester DNA Sequencing Facility. 25-100 clones were analyzed for each described experimental condition.

### Quantification and Statistical Analysis

#### Analysis of microarray data

Data were verified to be normally distributed. The raw data was uploaded into Agilent’s GeneSpring Software, normalized and fold changes calculated. For each group of animals the probes with an absolute 2-fold-change in mRNA expression between VC and fiber-exposed mice were included in subsequent analyses. These were subjected to ANOVA unequal variations test with Benjamini-Hochberg corrections. Significant 2-fold or more changes (p < 0.05) were subjected to hierarchical clustering with average linkage. The clustered heat-map was visualized using GeneSpring. The network pathways were identified using Ingenuity Pathways Analysis software (Ingenuity Systems, Redwood City, CA).

#### Quantification of Ki-67 and phospho-Histone H3 positive cells

Quantification of Ki-67 and phospho-Histone H3 positive cells was carried out by counting the number of positively stained cells in 5 random fields of view and dividing by the total number of cells (between 700-1000 cells per animal were counted, n = 3 per group). Data presented as mean ± SD. Statistical significance of data was estimated using two-tailed Student’s t test.

#### Quantification of DNA damage

Quantification of DNA damage was performed by using a standard curve and plotting the OD values versus the amount of positive control at each concentration point. Data presented as mean ± SD. Statistical significance of the data was estimated using a two-tailed Student’s t test.

#### Real time PCR

Quantification was performed using the comparative C_T_ method (ΔΔC_T_). Data presented as mean ± SD (n = 4 for each group). Statistical significance was assessed as p < 0.05 using two-tailed Student’s t test.

#### Relative quantification of gene copy number by real-time PCR

The ΔC_T_ score was calculated for each of the reactions using the following equation: C_T_ gene 1 − C_T_ gene 2 = ΔC_T_, where gene 1 is that of common DNA loss and gene 2 is the reference. The mean ΔC_T_ from the triplicate reactions was used for subsequent analysis. A series of four gDNA samples acted as control for each PCR run. The relative quantification of copy number was calculated using the 2^-ΔΔCT^ value. Z scores for the controls and predominantly p19 negative tumor areas were generated using the mean and SD of a reference population comprising the four control ΔC_T_ values. This was used to determine which samples were outside of the 99% reference range for control, with those outside of this range being considered aneuploid.

### Data and Software Availability

The dataset for mRNA array, the “gene changes in response to asbestos and carbon nanotube exposure in the pleural cavity, measured using microarrays,” reported in this paper is NCBI GEO: GSE51636 (https://www.ncbi.nlm.nih.gov/geo/query/acc.cgi?acc=GSE51636).

## Author Contributions

T.C. and F.A.M. designed experiments and co-wrote the paper. T.C., S. Galavotti, and F.A.M conducted the majority of experiments and prepared the manuscript. X.-M.S., I.R.P., S. Grosso, and A.S. conducted experiments and helped with manuscript preparation. A.N. and J.B. recruited patients and contributed to discussions. C.A.P. and K.D. contributed to experimental design, data interpretation, and writing of the paper. P.G., J.Z.-C., D.D., K.M.D., and J.L.Q. performed data analysis. M.M., T.C., A.E.W., and M.B. conceived the study, designed experiments, and wrote the manuscript.

## Supplemental Information

Document S1. Figures S1–S7 and Tables S1–S4

Document S2. Article plus Supplemental Information
