# Supplementary material for: Long-Fiber Carbon Nanotubes Replicate Asbestos-Induced Mesothelioma with Disruption of the Tumor Suppressor Gene Cdkn2a (Ink4a/Arf)
Source: Curr Biol. 2017 Nov 6;27(21):3302–3314.e6. doi: 10.1016/j.cub.2017.09.007 (PMC5681354; doi:10.1016/j.cub.2017.09.007)
Supplement: Document S1. Figures S1–S7 and Tables S1–S4 [file mmc1.pdf]

Current Biology, Volume *27*

Supplemental Information

Long-Fiber Carbon Nanotubes Replicate Asbestos-Induced Mesothelioma with Disruption of the Tumor Suppressor Gene *Cdkn2a* (*Ink4a/Arf*)

Tatyana Chernova, Fiona A. Murphy, Sara Galavotti, Xiao-Ming Sun, Ian R. Powley, Stefano Grosso, Anja Schinwald, Joaquin Zacarias-Cabeza, Kate M. Dudek, David Dinsdale, John Le Quesne, Jonathan Bennett, Apostolos Nakas, Peter Greaves, Craig A. Poland, Ken Donaldson, Martin Bushell, Anne E. Willis, and Marion MacFarlane

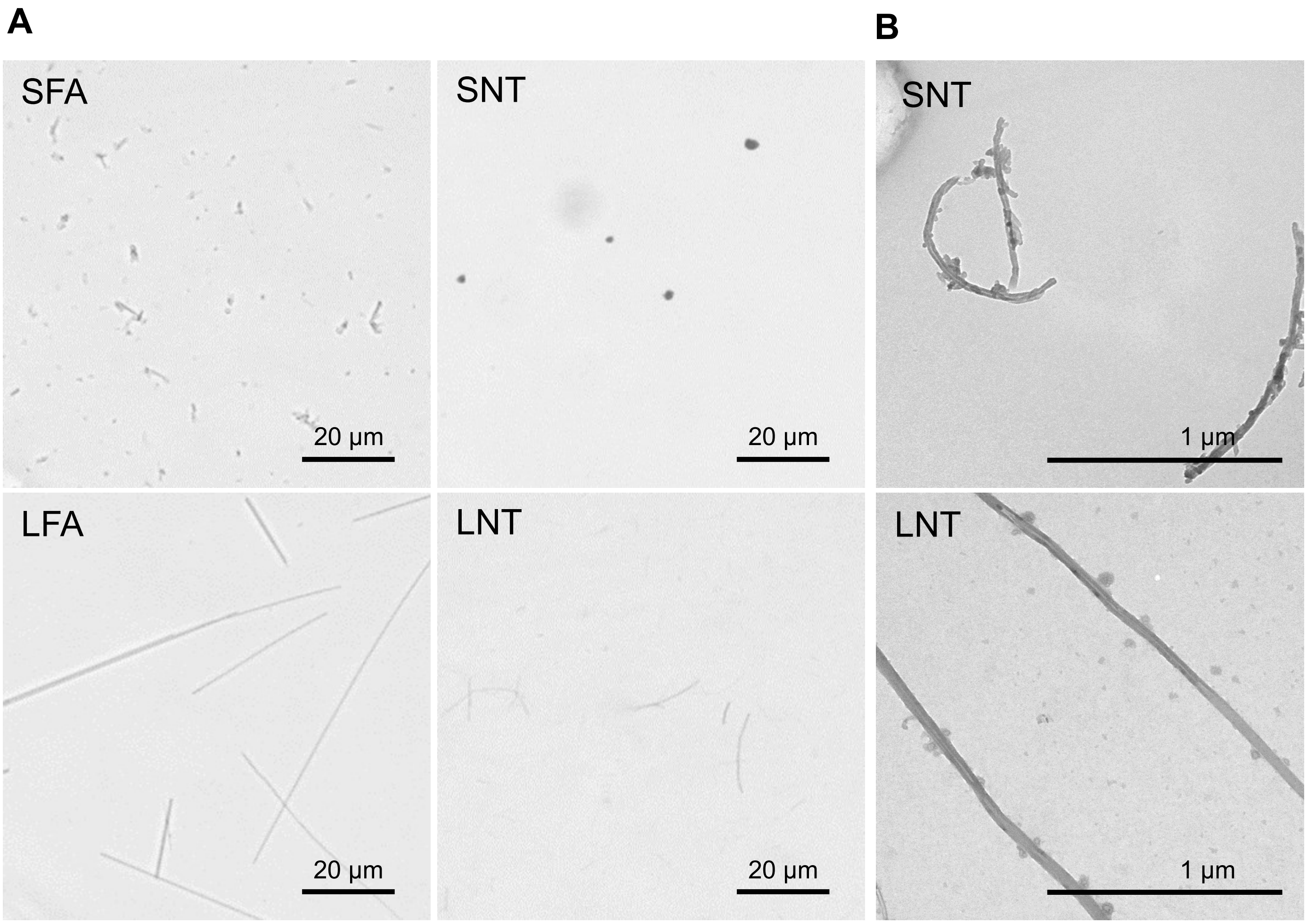

Figure S1, related to Figure 1. Dispersion of fibre panel for injection
A. Light micrographs of fibre panel as prepared to the standard degree of dispersion in 0.5% BSA used for intrapleural injection. For SNT we see a number of small respirable (>5 μm diameter) aggregates but no long fibres. Scale bars, 20 μm. **B.** TEM images of SNT and LNT samples after dispersion by ultrasonication. Scale bars, 1 μm.

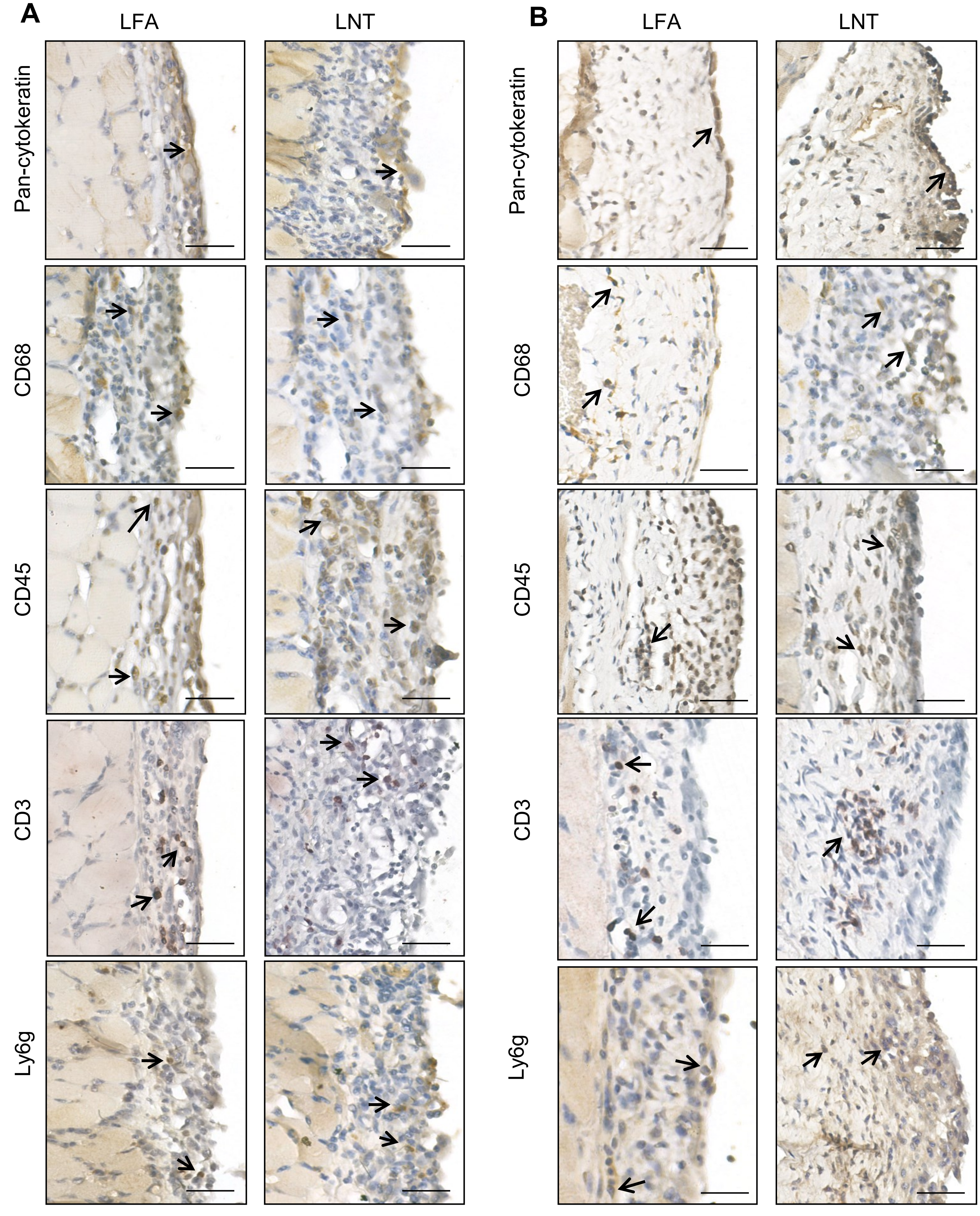

Figure S2, related to Figure 1. Sustained inflammatory response to long fibres at 1 and 12 weeks post-injection

A. Immunostaining of different cell types present in the lesions for a panel of cell markers at 1 week post-injection of LFA and LNT. **B**. Immunostaining of different cell types present in the lesions at 12 weeks post-injection. Black arrows indicate positive staining for each antibody (Pan-cytokeratin: mesothelial cells, CD68: macrophages, CD45: leukocytes, CD3: T-cells, Ly6g: granulocytes). Scale bars, 20 μm.

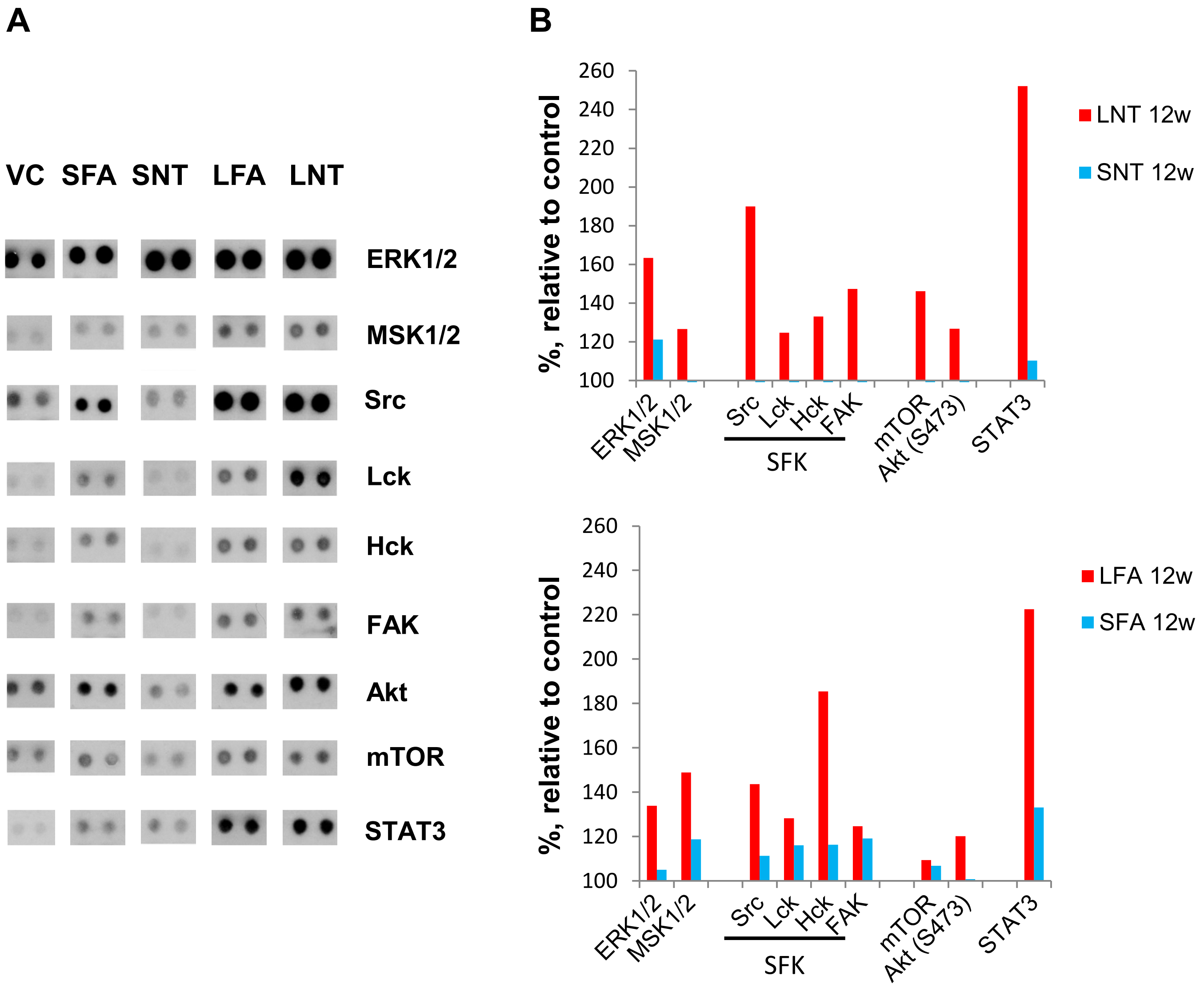

Figure S3, related to Figure 1. Kinome profiling of pleural tissue from fibre-exposed and control mice

A. A phosphokinase array was performed to screen the phosphorylation state of 64 kinases in the pleural tissue of control mice (VC) or mice exposed to SFA, LFA, SNT and LNT for 12 weeks. For the array, lysates from the pleurae of 4 animals per group were pooled. The image is an extract of the immunoblot of kinases activated in response to fibre exposure. LNT and LFA exposure caused marked and sustained stimulation of ERK1/2, Src and Src-family kinases (Lck, Hck and FAK), Akt and STAT3 signalling pathways. Exposure to SFA and SNT resulted in significantly less activation of these pathways. **B.** Kinase phosphorylation status was examined and those activated were compared in all groups of animals relative to VC (100%). Antibody array-based kinome profiling showed common sustained activation of pro-oncogenic pathways in mice exposed to long but not short CNT and asbestos fibres.

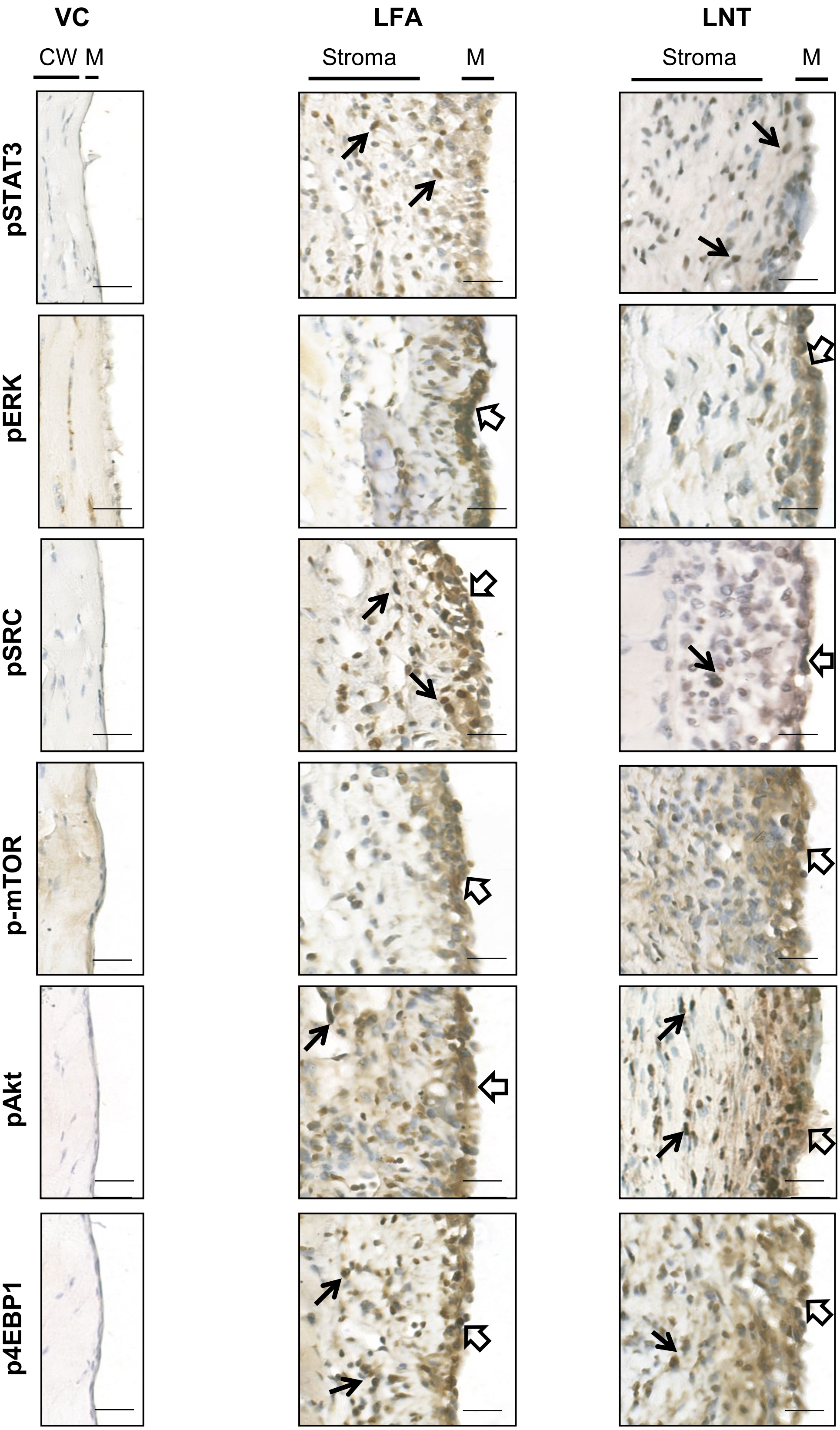

Figure S4, related to Figure 2. Contribution of cell types to aberrant signalling
Activation of pro-oncogenic signalling pathways in the chest wall (CW) tissue of mice 12 weeks post-injection was analysed *in situ* by immunohistochemistry. Black arrows indicate positive staining of inflammatory cells within the lesions; white arrows indicate positive staining in the mesothelial cells (M). Scale bars, 20 μm.

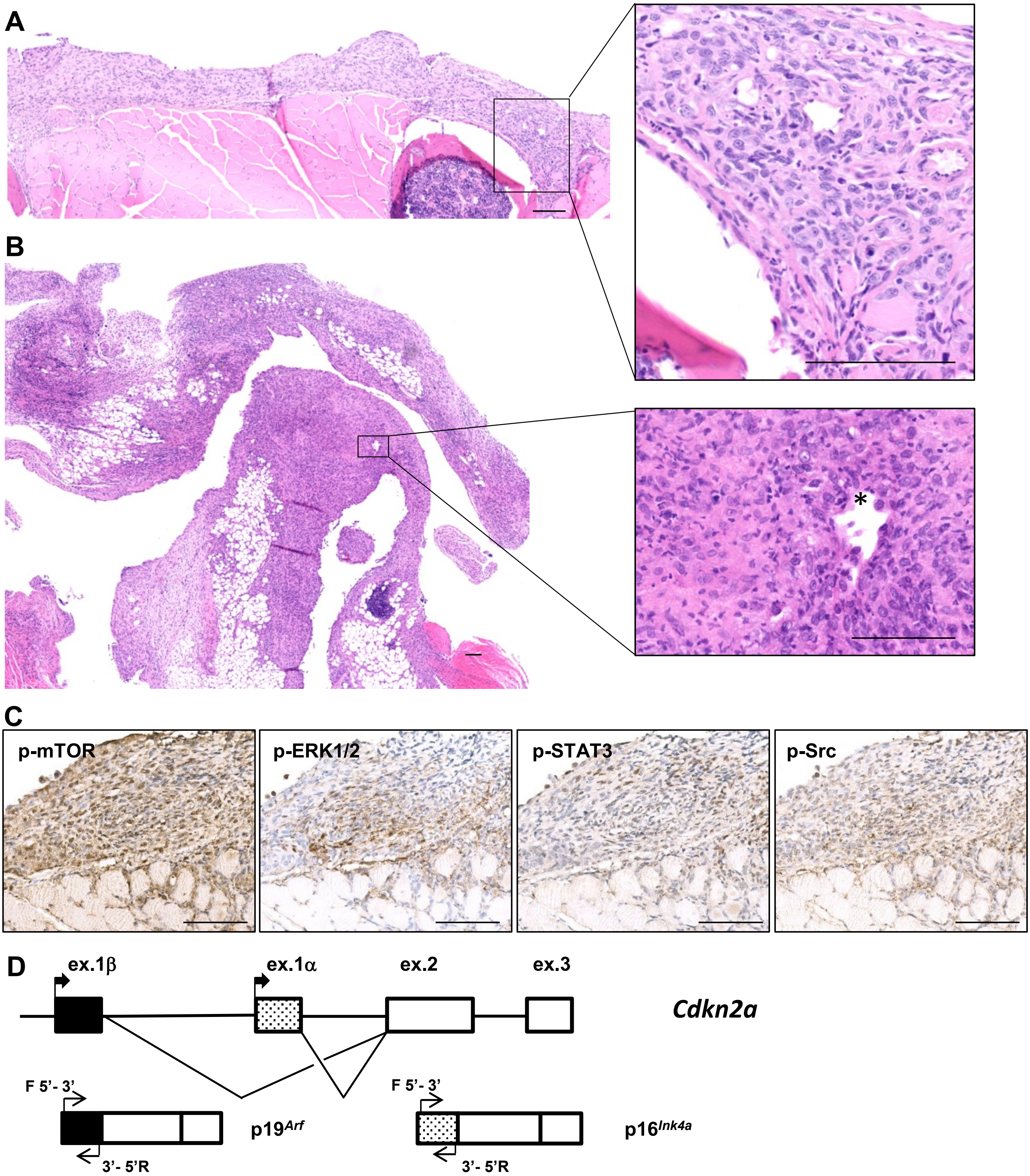

Figure S5, related to Figure 4. LNT-induced mesothelioma at 1 year post-injection exhibits pro-oncogenic signalling pathway activation

A. Haematoxylin and Eosin (H&E) images of the parietal pleura of the chest wall. High magnification displays pleomorphic tumour cells infiltrating into the underlying muscle of a mouse treated with 2.5 μg LNT (Animal ID #610). **B.** Tumour mass from pericardial soft tissue. High power call-out shows mass composed of pleomorphic tumour cells and lacunae lined by plump cells (*). **C.** Activation of pro-oncogenic signalling pathways in LNT-induced mesothelioma tissue was examined using phospho-specific antibodies. Scale bars, 100 μm. **D.** Schematic of primer design for p16^{Ink4a} and p19^{Arf} gene copy number quantification.

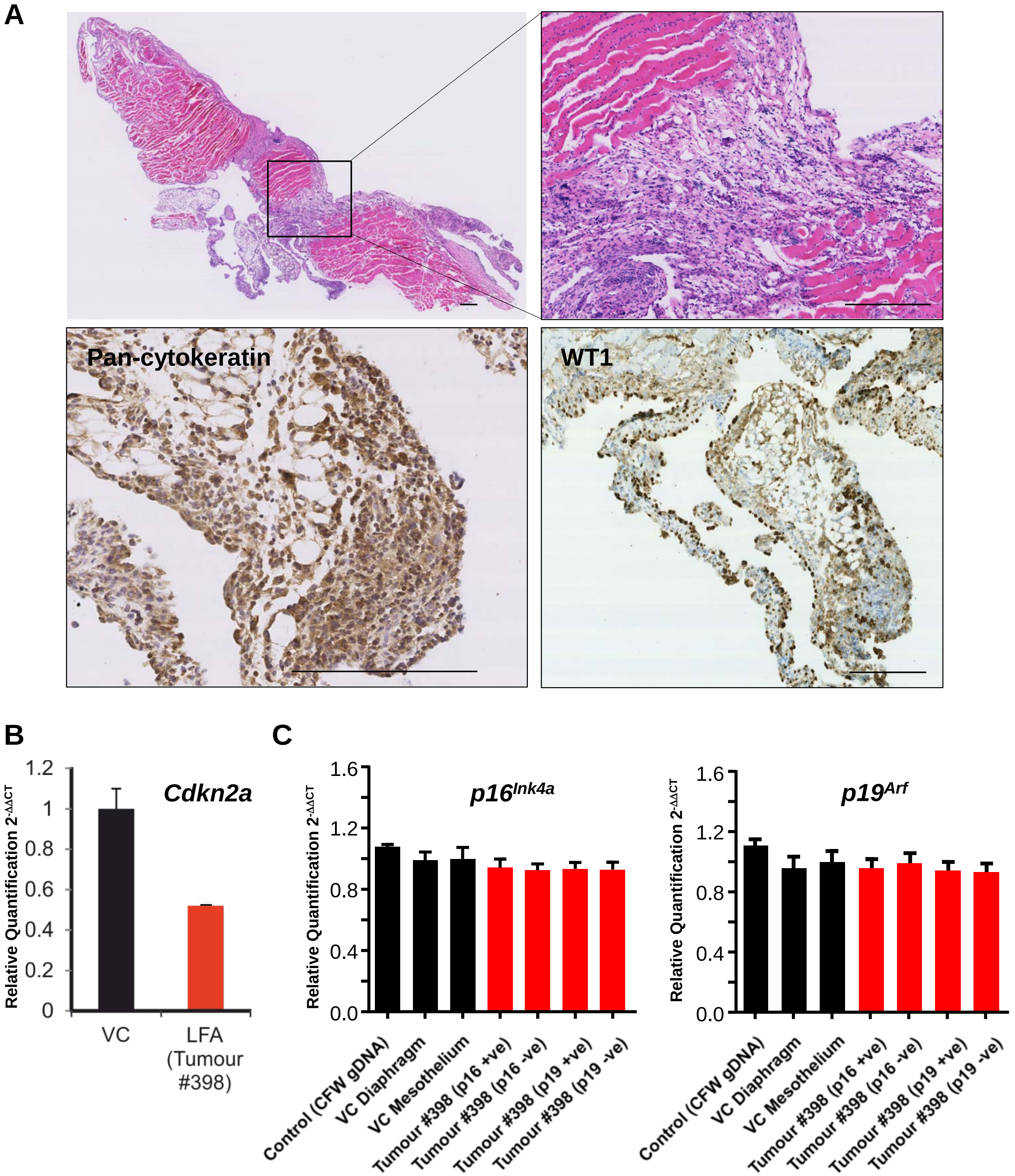

Figure S6, related to Figure 4. LFA-induced mesothelioma at 17 months post-injection
A. Haematoxylin and Eosin (H&E) images of mesothelioma on the diaphragm in LFA-treated mouse (Animal ID #398). High magnifications display pleomorphic tumour cells infiltrating into the underlying muscle tissue, positive for Pan-cytokeratin and WT1. Scale bars, 100 μm. **B.** *Cdkn2a* expression compared to control (VC, n=4) quantified by qPCR. **C.** Relative quantification (mean of $2^{-\Delta\Delta CT}$) of *p16* ^{Ink4a} and *p19* ^{Arf} gene copy number by qPCR analysis in gDNA isolated from positively- and negatively-stained areas in the LFA-induced tumour (Animal ID #398), compared to controls.

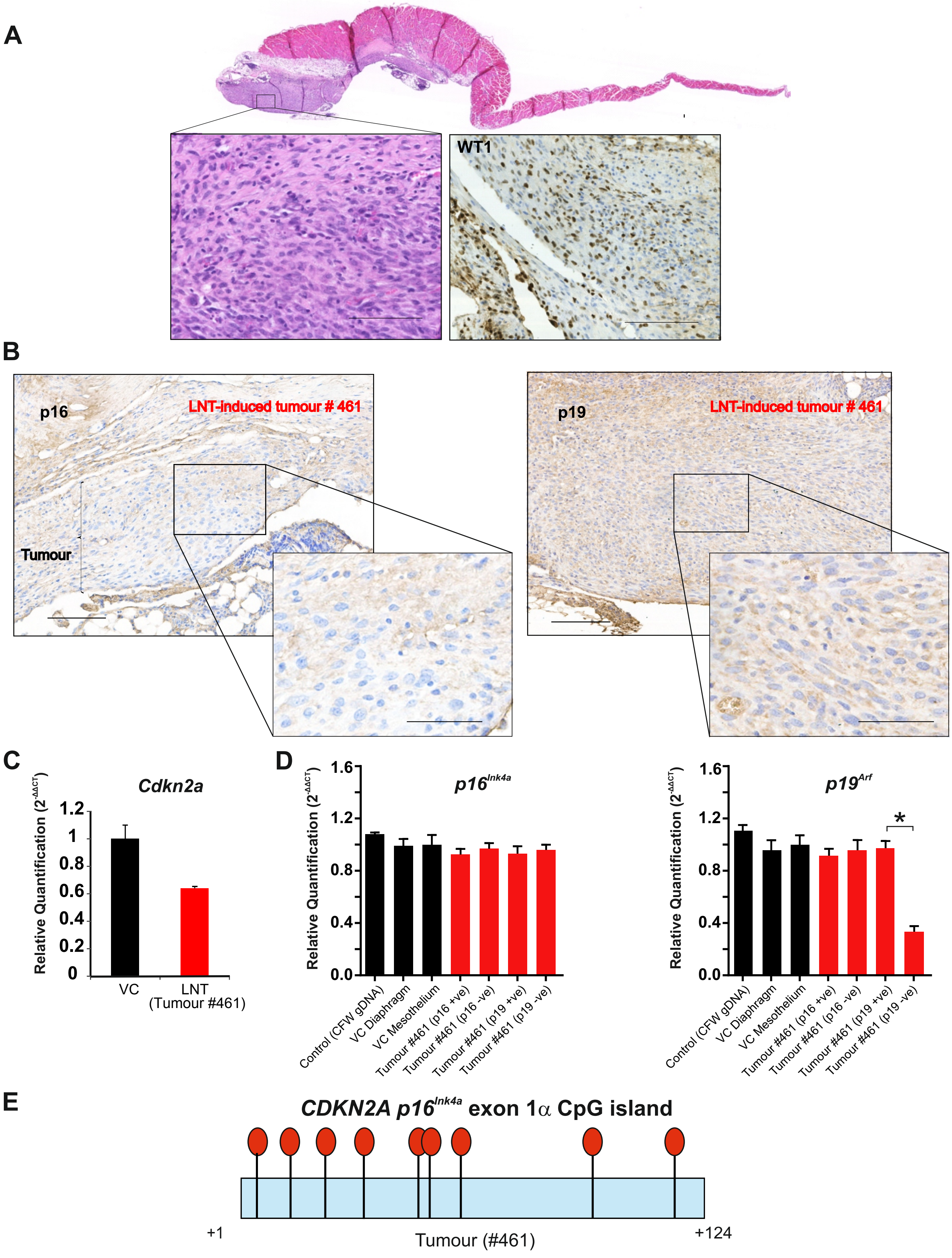

Figure S7, related to Figures 4 and 6. Low-dose LNT-induced mesothelioma at 17 months post-injection
A. Malignant mesothelioma of sarcomatoid type on the parietal pleura of mouse (Animal ID #461) treated with 1μg of LNT. High power call-outs show mass composed of tumour cells positive for WT1. **B**. Loss of p16 and p19 protein expression in tumour cells. Representative images of immunostaining, showing patchy pattern of p16 staining and predominantly negative staining for p19. Scale bars, 100 μm. **C**. *Cdkn2a* expression compared to contol (VC, n=3) quantified by qPCR. **D**. Relative quantification (mean of $2^{-\Delta\Delta CT}$) of *p16*^{Ink4a} and *p19*^{Arf} gene copy number by qPCR analysis in gDNA isolated from positively- and negatively-stained areas of the tumour compared to controls. Graphs show mean ± S.D., * $p < 0.05$ (significant difference is defined by Z score analysis, see also STAR Methods). **E.** Schematic representation of the hypermethylation profile of the *p16*^{Ink4a} CpG island in exon 1α, determined by bisulphite sequencing of gDNA from mesothelial cells. Hypermethylated CpG sites were identified in gDNA extracted from p16-negative areas of LNT-induced tumour (red-filled circles).

Table S1, related to Figure 1. Top networks identified in fibre-exposed mice at 12 weeks post-injection

	Associated Network Functions	Score
1	Inflammatory Response, Humoral Immune Response, Protein Synthesis	33
2	Cell Cycle, Cellular Movement, DNA Replication, Recombination, and Repair	31
3	Cellular Assembly and Organization, Cellular Function and Maintenance, Cellular Movement	27
4	Cell-To-Cell Signalling and Interaction, Haematological System Development and Function, Cellular Function and Maintenance	25

Significantly highly represented networks identified using Ingenuity Pathways Analysis software (Ingenuity® Systems, Redwood City, CA). Networks that were significantly highly represented ($P \leq 10^{-10}$; Fischer's exact test) were identified from the gene list, with significant difference (ANOVA) compared to VC, using Ingenuity Pathways Analysis software (Ingenuity Systems, Redwood City, CA). The network score describes the probability ($P=10^{-\text{network score}}$) that the molecules in the network are associated with the dataset by chance alone. Networks with a score of +30 were viewed as highly significant (10 = minimum score).

Table S2, related to Figure 1. Fibre source and characterization

	LFA	SFA	LNT	SNT
**Sample**	Long fibre amosite asbestos	Short fibre amosite asbestos	Long straight carbon nanotubes	Short straight carbon nanotubes
**Source**	Manville Corporation, USA	Manville Corporation, USA	University of Manchester, Dr. Ian Kinloch	Nanostructured and Amorphous Material Inc., USA
**Diameter (nm)**	1000	700	165	125
**% fibres >15 μm**	50	4	85	0

Table S3, related to Figure 1. Soluble metal contamination in aqueous extracts

Sample	Cd	Co	Cr	Cu	Fe	Mn	Ni	Ti	V	Zn
SFA	<0.1	2.1	<0.1	3.1	547	36.3	18.4	31.5	3.1	10.5
LFA	<0.1	1.4	3.4	5.2	853	104.8	5.1	2.0	<0.1	27.3
SNT	<0.1	<0.1	<0.1	<0.1	24.2	50.3	21.6	0.4	<0.1	5.3
LNT	<0.1	3.4	<0.1	1.2	37.3	3.6	6.2	0.3	<0.1	<0.1

The soluble metal contamination was assessed by preparing a soluble fraction from fibre samples suspended in distilled water and mixed at room temperature overnight. Fibres were removed by filtration. Metal concentration expressed as μg/g. The limit of detection by this method is 0.1μg/g. Prior to injection, the fibre samples were dispersed by ultrasonication in 0.5% bovine serum albumin (BSA; Sigma-Aldrich, Poole UK)/saline.

Table S4, related to STAR Methods section. Primer sequences

**CD68 F**	5’-GCGGTGGAATACAATGTGTC-3’
**CD68 R**	5’-TGGAGCTCTCGAAGAGATGA-3’
**Mesothelin F**	5’-ACAAATGGACCTTGTGAACG-3’
**Mesothelin R**	5’-CTGGATCAGGGACTCAGGAT-3’
**IL-6 F**	5’-ACAAAGCCAGAGTCCTTCAGAGA-3’
**IL-6 R**	5’-CTGTTAGGAGAGCATTGGAAATTG-3’
**PI3K F**	5’-GATGGCGAAGAGCTAGGTAAGC-3’
**PI3K R**	5’-GATGGCGAAGAGCTAGGTAAGC-3’
**STAT3 F**	5’-TCGTGGAGCTGTTCAGAAAC-3’
**STAT3 R**	5’-TGACCAGCAACCTGACTTTC-3’
**CDKN2A F**	5’-AGACCGACGGGCATAGCTT-3’
**CDKN2A R**	5’-TAGCTCTGCTCTTGGGATTGG-3’
**p16BSF3**	5’-AGTGTTTTCAGGGGTGTTCAATTCAT-3’
**p16ZSF2**	5’-TATTTTTAGAGGAAGGAAGGAGGGATT-3’
**p16REZSg**	5’-CCAATCTATCTACAACGAACTCCA-3’
**p16REZSa**	5’-CCAATCTATCTACAACAAACTCCA-3’
**p16ZS Rg**	5’-CAAAAATTACCCGACTACAAATAAAACACTCCTTACC-3’
**p16ZS Ra**	5’-CAAAAATTACCCAACTACAAATAAAACACTCCTTACC-3’
**PR19F1c**	5’-GTCGATTTTTTTATTTTTTTTTTTTTATAGATG-3’
**PR19F1t**	5’-GTTGATTTTTTTATTTTTTTTTTTTTATAGATG-3’
**PR19F2c**	5’-GAAAGGGCGTAGTTATTGTTAGGGGG-3’
**PR19F2t**	5’-GAAAGGGTGTAGTTATTGTTAGGGGG-3’
**PR19 R1a**	5’-CAAAAAAACTTCCCAAAAAC-3’
**PR19 R1g**	5’-CGAAAAAACTTCCCAAAAAC-3’
**p16 F**	5’-TCACACGACTGGGCGATT-3’
**p16 R**	5’-GGACTCCATGCTGCTCCA-3’
**p16 probe**	5’-FAM-GCGAGGAAAGCGAA-MGB-3’
**p19 F**	5’-TGGTGAAGTTCGTGCGAT-3’
**p19 R**	5’-GCCCTCTTCTCAAGATCCTCT-3’
**p19 probe**	5’-FAM-CTAGCCTCAACAACATGTT-MGB-3’
**Svil F**	5’-ACCTGCCCACTCAGAGAACT-3’
**Svil R**	5’-TGTGTGACTGGTCAATGTGG-3’
**Svil probe**	5’-VIC-ATACAGGAGCATTTCACTC-MGB-3’
**β2m F**	5’-CATACGCCTGCAGAGTTAAGCA-3’
**β2m R**	5’-GATCACATGTCTCGATCCCAGTAG-3’
**M13**	5’-CAGGAAACAGCTATGAC-3’
